# Supplementary material for: Impact of a culturally tailored parenting programme on the mental health of Somali parents and children living in Sweden: a longitudinal cohort study
Source: BMJ Open. 2021 Aug 19;11(8):e045067. doi: 10.1136/bmjopen-2020-045067 (PMC8378386; doi:10.1136/bmjopen-2020-045067)
Supplement: Supplementary data [file bmjopen-2020-045067supp001.pdf]

Supplementary

Supplemental Material for

The impact of a culturally tailored parenting program on the mental health of Somali parents and children living in Sweden: A longitudinal cohort study

Fatumo Osman[a,c], Linda Vixner[a], Renée Flacking[a], Marie Klingberg-Allvin[a], Ulla-Karin Schön[b] & Raziye Salari[c]

[a] *School of Education, Health and Social Studies, Dalarna University, Falun, Sweden*

[b] *Department of Social Work, Stockholm University, Stockholm, Sweden*

[c] *Child Health and Parenting (CHAP), Department of Public Health and Caring Sciences, Uppsala University, Uppsala, Sweden*

Supplementary

Table S1.

Content of the intervention: Connect program and societal information

Session	Intervention topics	Content of the session
**Societal information**		
1	Child Welfare Services	- Knowledge and understanding on social service's work with children and youngsters.<br>- Various laws in the social services regarding the removal of the child from his/her family<br>- The support that social services have to offer for families
1	Parenting styles; authoritarian and democratic parenting	- Different parenting styles and the impact it has on child development<br>- Knowledge and understanding of parental responsibility regarding personal, economic and social growth<br>- Interaction between children and parents in the new context
2	Convention on the rights of the child	- Knowledge on the United Nations Convention on the Rights of the Child<br>- Reflection on how parents follow the child's right<br>- Reflection on having a child perspective
- Connect program		
3	Introduction on Connect parenting program	- Introduction to Connect and the theory that the program is based on
4	All behaviour has a meaning	Children's behaviour and what it stands for, and how parents could recognize it
5	Attachment is for life	Skills to recognize the attachment needs of the infant, small children and teens
6	Conflict is a part of attachment	Conflict as a natural part of the relationship between parents and teens
7	Autonomous includes connection	The teens need regarding the autonomous while they want to be connected with their parents.
8	Empathy – the heartbeat of attachment	Practice and acknowledge empathy which is about being there for the child without condemning or giving solution of the problem.

Supplementary

9	Balance our needs with needs of others	Parents to recognize their own attachment needs and look for other sources of support than their children.
10	Growth and change are part of relationship	Parents to become aware of their past, and acknowledge what can promote and hinder the teens change and growth.
11	Celebrating attachment	Acknowledgement of that in every relationship there are both joy and pain which should be celebrated.
12	Two steps forward, One step back: staying on course	Understand on how to respond when the relationship is on turbulence and how doing that can establish a reconciliation and strengthen the relationship.

Supplementary

Table S2.

Internal consistency (Cronbach's alpha) for outcome measures at baseline, two-month follow-up, and three-year follow-up (N = 40-51)

	Number of items	Cronbach's alpha		
		Baseline	2-month FU	3-year FU
**CBCL 6-18**				
Total problem score	99	.82	.75	.69
Internalizing problems	32	.29	.62	.43
Externalizing problems	33	.85	.73	.75
Social problems	11	.29	.30	0
Thought problems	13	-.03	.21	.36
Attention problems	10	.75	.54	-.02
**GHQ-12**[a]	12	.80	.91	.81

CBCL 6-18 = Child Behaviour Checklist for children 6-18 years, GHQ = General Health Questionnaire; FU = follow-up

As shown in Table S2, the Cronbach's alpha for internalizing problems and the three syndrome scales were low and thus no further analyses were reported on these outcomes in the main paper. Below, we report the analyses for all the outcomes regardless of the level of Cronbach's alpha.

RESULTS

Long-term findings

Although most variables were not normally distributed, ANOVA is considered relatively robust to violations of normality assumptions [36, 37]

Mauchly's test indicated that the assumption of sphericity had been violated for five outcomes (CBCL total problem scores, externalizing, social problems and attention problems as well as GHQ-12), therefore following the recommendations by Howell, [38] we report multivariate tests (Pillai's Trace) for these five outcomes. The results show significant improvement over time for all outcomes (see Table S3 (compatible to Table 2 in the main paper) for means, standard deviations, mean differences, *F* values, and 95% confidence intervals [*CI*s] and effect sizes).

For parent-reported CBCL, repeated measure ANOVA revealed significant improvements in children for all primary outcomes from baseline to the three-year follow-up: total problem scores (95% *CI*, 11.49–18.00), internalizing problems (95% *CI*, 4.43–6.24), and externalizing problems (95% *CI*, 2.48–5.83). The effect sizes were all large. Similarly, significant improvements were observed for all secondary outcomes, i.e., CBCL scores for social, thought, and attention problems in children, and parents' mental health as measured by GHQ-12. The associated effect sizes for the secondary outcomes were medium to large.

Supplementary

Table S3.

Within-group comparison of intervention participants at baseline, two-month follow-up, and three-year follow-up (N = 51)

					Within-subject change							
					Baseline to 2-month FU				Baseline to 3-year FU			
	Baseline Mean (*SD*)	2-month FU Mean (*SD*)	3-year FU Mean (*SD*)	*F* (2, 49) [a]	Mean diff	*F* (1, 50) [b]	95% *CI*	*d*	Mean diff	*F* (1, 50) [b]	95% *CI*	*d*
**CBCL 6-18**												
Total problem score	15.71 (9.8)	9.49 (7.4)	0.96 (2.1)	95.86***	6.22	15.13***	2.26-10.18	0.54	14.75	125.84***	11.49-18.00	1.57
Internalizing problems	5.65 (2.6)	3.76 (2.9)	0.31 (0.8)	84.08***	1.88	15.98***	0.72-3.05	0.56	5.33	212.50***	4.43-6.24	2.04
Externalizing problems	4.67 (5.2)	2.39 (3.5)	0.51 (1.4)	26.94***	2.28	8.28*	0.32-4.23	0.40	4.12	37.77***	2.48-5.83	0.86
Social problems	1.65 (1.5)	0.76 (1.2)	0.02 (0.1)	35.95***	0.88	12.78**	0.27-1.49	0.50	1.63	61.44***	1.11-2.14	1.10
Thought problems	0.61 (0.8)	0.53 (0.9)	0.08 (0.3)	9.83***	0.08	0.29	-0.28-0.44	0.08	0.53	23.27***	0.26-0.80	0.68
Attention problems	1.23 (2.2)	0.61 (1.3)	0.04 (0.2)	11.80***	0.63	3.72	-0.18-1.43	0.27	1.20	15.59***	0.45-1.95	0.55
**GHQ-12**[a]	20.00 (4.2)	17.80 (4.8)	18.22 (2.3)	5.46**	2.18	7.37*	0.19-4.18	0.41	1.76	10.28**	0.40-3.11	0.46

[a] *F*(2, 47) for GHQ-12

[b] *p* values adjusted for multiple comparison (Bonferroni); *F*(1, 48) for GHQ-12

CBCL 6-18 = Child Behavior Checklist for children 6-18 years, GHQ = General Health Questionnaire; FU = follow-up

Grey marked indicate outcomes with low Cronbach's alpha

*** $p < .001$; ** $p < .01$; * $p < .05$

Supplementary

Clinical significance of change in children's and parents' mental health

Figure S1 shows the clinical significance of change in the mental health of children and parents at two-month and three-year follow-ups. The proportion of children who exhibited no clinically significant change from baseline to the two-month follow-up ranged from 61% on social problems to 88% on attention problems. This indicated that the level of various problems remained unchanged for most children. Very few children exhibited negative changes (4% to 8%), while positive changes were observed for a higher number of children (8% to 31%).

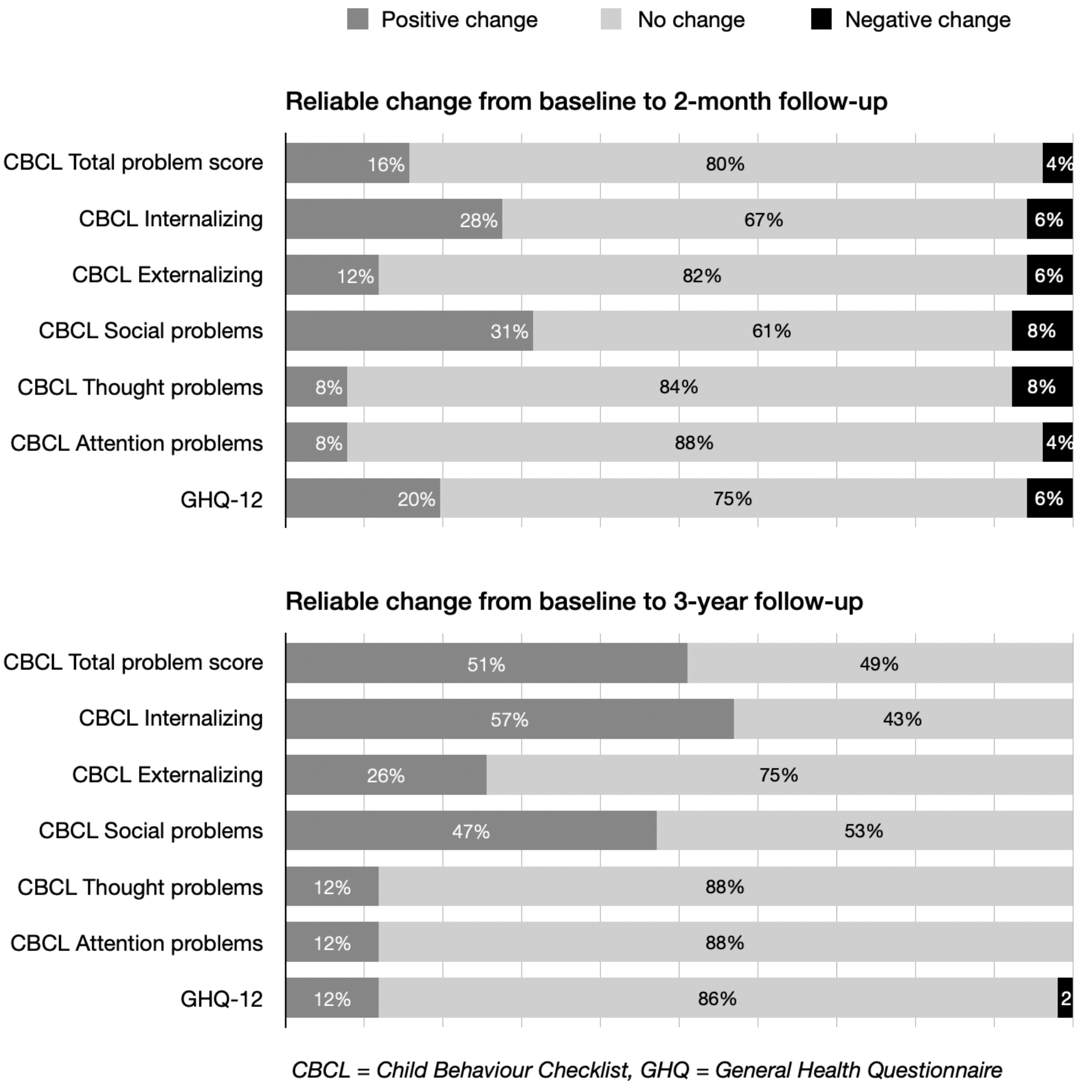

Figure S1. Clinical significance of change from baseline: proportion of children (CBCL) and parents (GHQ) showing reliable change (N =51 for children and 49 for parents)

Supplementary

The positive changes were more pronounced from baseline to the three-year follow-up: 25% to 57% of children demonstrated clinically significant improvement on the primary outcomes, while 12% to 47% demonstrated clinically significant improvement on the secondary outcomes. No negative changes were observed for children in any of the outcomes.

Most parents showed no clinically significant changes from baseline to the two-month or three-year follow-ups (71% and 82%, respectively). Very few showed negative changes (6% and 2% at the two-month and three-year follow-ups, respectively), while positive changes were observed for a higher number of parents (20% and 12% at the two-month and three-year follow-ups, respectively).

Supplementary and sensitivity analyses. Long-term follow-up data were available for 36 out of 39 parents in the control group who had received the intervention. One-way repeated measure ANOVAs on this group revealed similar significant improvement from baseline to the long-term follow-up for all the outcomes except CBCL attention problems, which had remained the same. In addition, we imputed the data for the six participants who had dropped out from the study using the worst available scores (i.e., worst case scenario). One-way repeated measure ANOVAs on the imputed data revealed similar significant improvement from baseline to the long-term follow-up for all the outcomes. These results point to the robustness of the main findings.

REFERENCES

36. Harwell M.R., et al., Summarizing Monte Carlo results in methodological research: The one- and two-factor fixed effects ANOVA cases. *J. Educ. Stat.* 1992. 17(4): 315-339.
37. Lix, L.M., J.C. Keselman, and H.J. Keselman, Consequences of assumption violations revisited: A quantitative review of alternatives to the one-way analysis of variance "F" test. *Rev. Educ. Res.* 1996. 66(4): 579-619.
38. Howell, D.C. (2009). *Statistical Methods for Psychology* (5th ed.). Pacific Grove CA: Duxbury.
