# Supplementary material for: Impact of a culturally tailored parenting programme on the mental health of Somali parents and children living in Sweden: a longitudinal cohort study
Source: BMJ Open. 2021 Aug 19;11(8):e045067. doi: 10.1136/bmjopen-2020-045067 (PMC8378386; doi:10.1136/bmjopen-2020-045067)
Supplement: Author's manuscript [file bmjopen-2020-045067.draft_revisions.pdf]

BMJ Open is committed to open peer review. As part of this commitment we make the peer review history of every article we publish publicly available.

When an article is published we post the peer reviewers' comments and the authors' responses online. We also post the versions of the paper that were used during peer review. These are the versions that the peer review comments apply to.

The versions of the paper that follow are the versions that were submitted during the peer review process. They are not the versions of record or the final published versions. They should not be cited or distributed as the published version of this manuscript.

BMJ Open is an open access journal and the full, final, typeset and author-corrected version of record of the manuscript is available on our site with no access controls, subscription charges or pay-per-view fees (http://bmjopen.bmj.com).

If you have any questions on BMJ Open's open peer review process please email info.bmjopen@bmj.com

BMJ Open

Long-term outcomes of a parenting program on Somali parents' and their children's mental health

Journal:	*BMJ Open*
Manuscript ID	bmjopen-2020-045067
Article Type:	Original research
Date Submitted by the Author:	21-Sep-2020

Complete List of Authors:	Osman, Fatumo; Hogskolan Dalarna, School of Education, Health and Social Studies; Uppsala University, Department of Public Health and Caring Sciences<br>Vixner, Linda; Hogskolan Dalarna, School of Education, Health and Social Studies<br>Flacking, Renee; Hogskolan Dalarna, School of Education, Health and Social Studies<br>klingberg-allvin, marie; Högskolan Dalarna, School of Education, Health and Social Studies<br>Schön, Ulla-Karin; Stockholm University, Department of Social Work<br>Salari, Raziye; Uppsala Universitet Institutionen for folkhalso- och vardvetenskap, Child Health and Parenting, Department of Public Health and Caring Sciences

Keywords:	MENTAL HEALTH, PUBLIC HEALTH, Child & adolescent psychiatry < PSYCHIATRY, Community child health < PAEDIATRICS

SCHOLARONE™ Manuscripts

I, the Submitting Author has the right to grant and does grant on behalf of all authors of the Work (as defined in the below author licence), an exclusive licence and/or a non-exclusive licence for contributions from authors who are: i) UK Crown employees; ii) where BMJ has agreed a CC-BY licence shall apply, and/or iii) in accordance with the terms applicable for US Federal Government officers or employees acting as part of their official duties; on a worldwide, perpetual, irrevocable, royalty-free basis to BMJ Publishing Group Ltd ("BMJ") its licensees and where the relevant Journal is co-owned by BMJ to the co-owners of the Journal, to publish the Work in this journal and any other BMJ products and to exploit all rights, as set out in our licence.

The Submitting Author accepts and understands that any supply made under these terms is made by BMJ to the Submitting Author unless you are acting as an employee on behalf of your employer or a postgraduate student of an affiliated institution which is paying any applicable article publishing charge ("APC") for Open Access articles. Where the Submitting Author wishes to make the Work available on an Open Access basis (and intends to pay the relevant APC), the terms of reuse of such Open Access shall be governed by a Creative Commons licence – details of these licences and which Creative Commons licence will apply to this Work are set out in our licence referred to above.

Other than as permitted in any relevant BMJ Author's Self Archiving Policies, I confirm this Work has not been accepted for publication elsewhere, is not being considered for publication elsewhere and does not duplicate material already published. I confirm all authors consent to publication of this Work and authorise the granting of this licence.

For peer review only

Long-term outcomes of a parenting program on Somali parents' and their children's mental health

Fatumo Osman[a,c], Linda Vixner[a], Renée Flacking[a], Marie Klingberg-Allvin[a], Ulla-Karin Schön[b] & Raziye Salari[c]

[a] *School of Education, Health and Social Studies, Dalarna University, Falun, Sweden*

[b] *Department of Social Work, Stockholm University, Stockholm, Sweden*

[c] *Child Health and Parenting (CHAP), Department of Public Health and Caring Sciences, Uppsala University, Uppsala, Sweden*

Corresponding author: Fatumo Osman, Dalarna University, 79188 Falun, Sweden, Telephone work: +4623778220, Cell phone: +46701918604, Email: fos@du.se

For peer review only

ABSTRACT

Objectives: The aim of this study was to conduct a three-year follow-up report on the effectiveness of a culturally tailored parenting support program (Ladnaan) for Somali-born parents living in Sweden.

Methods: This study is a long-term (three-year) follow-up of the intervention group (n=60) in a randomized controlled trial targeting Somali-born parents. The Ladnaan intervention consisted of two main components: societal information, and the Connect parenting program delivered using a culturally sensitive approach. The intervention included 12 group-based sessions. The primary outcome was improved mental health in children, as measured by the Child Behavior Checklist (CBCL). The secondary outcome was improved mental health in parents, as measured by the General Health Questionnaire (GHQ-12).

Results: Of the 60 parents who were originally randomized to the intervention group after the baseline assessment, 51 were included in this long-term follow-up. The one-way repeated measures (baseline to the three-year follow-up) Analysis of Variance (ANOVA) for the CBCL confirmed maintenance of all treatment gains for children: total problem scores (95%CI, 11.49–18.00, d=1.57), internalizing problems (95%CI, 4.43–6.24, d=2.04), and externalizing problems (95%CI, 2.48–5.83, d=0.86). Similar results were observed for parent mental health (95%CI, 0.40–3.11, d=0.46).

Conclusion: Positive changes in the mental health of Somali-born parents and their children were maintained three years after they had received a parenting support program that was culturally tailored and specifically designed to address their needs. Our findings highlight the long-term potential benefits of these programs in tackling the mental health issues in immigrant families.

For peer review only

Clinical Trial: This trial has been registered at www.clinicaltrials.gov (identifier NCT02114593).

For peer review only

ARTICLE SUMMARY

Strengths and limitations of the study

- The study design was a long-term (three-year) follow-up of an intervention group who received a culturally tailored parenting program.
- The majority of the original sample were retained in this three-year follow-up.
- The main limitation of the study was the lack of a control group, which only allowed within-group analyses.
- The control group was offered the intervention shortly after the two-month follow up due to ethical and practical reason.
- Data were collected through parent report.

For peer review only

INTRODUCTION

Over the past few decades, Sweden and many other European countries have experienced a rapid increase in immigration, particularly immigrants who have fled from areas of war and conflict. [1] Stress factors due to immigration have a negative influence on the mental health of parents and their children. [2-7] More specifically, these post-migration factors include a lack of social networks and support, perceived discrimination, inadequate accommodation, and acculturation challenges. [7-13] Studies have reported that the mental health problems of immigrant parents are associated with low confidence in parenting, [14-16] and the use of authoritarian parenting, which has been shown to be related to children externalizing problems. [6, 17, 18]

Parents with an immigrant background have reported a need for culturally tailored parenting support programs that assist them in adapting their parenting to the host country, improving their relationships with their children, [7, 19-22] and improving their children's mental health. [23] It has been demonstrated that parenting support programs that are culturally tailored to immigrant parents' needs improve children's mental health, strengthen parenting roles, and enhance parent-child relationships in the short-term. [24-27] However, little is known about the long-term effects of these programs on the mental health of parents and their children.

Objectives

The aim of this study was to report on a three-year follow-up of the impact of a culturally tailored parenting support program on the mental health of Somali-born parents and their children.

For peer review only

METHODS

Study design

This study is a long-term (three-year) follow-up of the intervention group in a randomized controlled trial (RCT), [25, 26] which evaluated the impact of a culturally tailored parenting support program for Somali-born parents living in Sweden, referred to as the Ladnaan intervention. Ethical approval was obtained from the Swedish Regional Ethical Review Board in Uppsala, Sweden (Dnr 2014/048/2), and the trial was registered at www.clinicaltrials.gov (identifier NCT02114593).

Originally, the study included a two-month as well as a six-month post-intervention follow-up. However, the participating municipality started offering the intervention to the control group shortly after the two-month follow-up data collection. As we had lost most of our control group by six months, we opted for a three-year post-intervention follow-up instead.

Setting and participants

The study was conducted in a town in the middle of Sweden with approximately 50,000 inhabitants, approximately 3,000 of whom are of Somali origin. Baseline (pre-intervention) and two-month follow-up data were collected between May 2014 and October 2015. The three-year post-intervention data were collected between April and October 2018. Data were collected by the first author and by research assistants who were trained in the recruitment process, taking informed consent, and use of the instruments in the study.

The main trial included 120 Somali-born parents with children aged 11–16 years and self-perceived stress related to parenting practices. If parents had more than one child aged 11-16, they were asked to complete the questionnaire for the child with whom they particularly wished to improve their relationship. In two-parent families, both parents were offered the

For peer review only

possibility to participate in the intervention, but only the parent who was screened at baseline completed the questionnaires throughout the study. Originally, we had planned to collect data also from the children themselves. However, parents did not consent to their children's direct participation in the study (mainly due to the fear of Social Services). Thus, only parent report data was available.

Baseline data was collected prior to randomization and the follow up data was collected two months as well as three years post-intervention. Compared to parents in the control group, parents in the intervention group reported significant improvement in their children's and their own mental health at the two-month follow-up. [25, 26] Since most of the parents in the control group received the intervention soon after the two-month follow up, the present paper focuses on the three-year follow-up of the original intervention group. Of the 60 parents in the intervention group who completed the baseline assessment, three were lost to the two-month follow-up, and six were lost to the three-year follow-up. Thus, 51 parents in the intervention group were followed up three years post-intervention (see Figure 1).

[Insert Figure 1 here]

Intervention

The Ladnaan intervention, developed based on a qualitative study conducted prior to the trial, [7] consisted of two main components: societal information and the Connect parenting program. [28, 29] It included 12 weekly sessions with groups of 12-17 parents, each lasting approximately 1-2 hours.

The first two sessions covered societal information relevant to Somali-born parents, including an overall view of the Swedish Child Welfare Services, the convention on the Rights of the Child, and parenting styles in Sweden. The other 10 sessions covered the Connect parenting support program, [28] which is a standardized program based on attachment theory including

For peer review only

role-plays, case examples, and reflection exercises. The cultural sensitivity delivery approach was employed to ensure the sustainability of the parenting program after the study was concluded. This delivery approach incorporated elements such as delivering the program in the participants' native language, using group leaders of similar background and experience, and culturally tailoring role-plays and case examples to make them understandable and relatable for the participants. Only the parenting style theme in the societal information sessions was delivered by a Swedish-speaking professional from the Family and Child Welfare Services and interpreted by one of the group leaders.

A detailed description of the intervention and the findings from the randomized controlled trial after the two-month follow-up is published elsewhere. [25, 26]

Outcomes

The *primary outcomes* in this study were the CBCL scores for the total, internalizing, and externalizing problems. The *secondary outcomes* were CBCL scores on symptoms of social, thought, and attention problems as well as the GHQ-12 scores.

The Child Behavior Checklist (CBCL) for children aged 6 to 18 years (CBCL 6–18) [30] was used to measure children's mental health from the parents' perspective. We opted to use the CBCL 6-18 even for the children who had already turned 18 at the three-year follow-up because (1) all the children were still living at home with their parents, (2) using the adult version would have led to difficulties in estimating changes in the level of problems from baseline. The CBCL 6–18 consists of 133 items encompassing two sections: (1) competence scales (20 items on activities and social scales), and (2) emotional and behavioral problems (113 items). In this study, only items related to emotional and behavioral problems were used. According to Achenbach System of Empirically Based Assessment (ASEBA) guideline, [30] alcohol- and sex-related items can be excluded for Muslim respondents. In this study, five

For peer review only

sex-related items were considered culturally inappropriate for our target population and thus omitted, leaving 107 items on CBCL. There were three possible response options for each problem item: 0 (not true), 1 (somewhat or sometimes true), and 2 (very true or often true). Using the ASEBA manual for CBCL 6–18, [30] we constructed six variables: a total problem score, two broad groupings of emotional and behavioral problems (internalizing and externalizing problems), and three syndrome scales (social, thought, and attention problems). Higher scores indicate greater problem severity.

The 12-item version of the General Health Questionnaire (GHQ-12) [31] was used to measure parents' overall mental health over the past few weeks. Each item was rated on a four-point Likert scale from 1 (better than usual) to 4 (much worse than usual), with higher scores indicating higher mental health distress.

Both CBCL 6–18 and GHQ-12 were translated into Somali following the five steps of the World Health Organization's process of translation. [32]

Statistical methods

All analyses were performed using IBM SPSS Statistics (Version 24). Descriptive statistics of baseline data were presented with mean (SD) or proportions (%). One-way repeated measure Analysis of Variances (ANOVAs) were used to compute the within-subject change over time from baseline to two-month follow-up and three-year follow-up for parents receiving the Ladnaan intervention. Cohen's d effect size was calculated from F tests ($d = 0.2$, small; $d = 0.5$, medium; and $d = 0.8$, large). [33]

In addition, the clinical significance of change was measured from baseline to two-month follow-up and three-year follow-up using the method recommended by Jacobson and Truax. [34] Population norms for the CBCL 6–18 and GHQ-12 were not available for the present study population. Therefore, we used the pretest scores for all participants in the original

For peer review only

randomized controlled trial to calculate the standard error of differences (Sdiff). We assumed a measurement reliability of 0.8 for each measure. The clinical significance of change was then presented as the proportion of children/parents who had deteriorated, remained unchanged, or improved at two-month and three-year follow-ups.

For peer review only

RESULTS

Attrition and baseline characteristics

Table 1 presents baseline characteristics of the participants who remained in the study at the three-year follow-up and those who dropped out. No statistically significant differences were found between the two groups. Of the 51 participants who remained at follow-up and were included in the analyses, 34 were mothers and 17 were fathers. Only five of them were employed at the time. Most of them had not entered upper secondary school, were married and living with their partner. The six participants who dropped out of the study at the three-year follow-up were all mothers, were educated up to upper secondary school level only, and were unemployed. Five of these six were not cohabiting with a partner.

[Insert Table 1 here]

Long-term findings

Mauchly's test indicated that the assumption of sphericity had been violated for five outcomes (CBCL total problem scores, externalizing, social problems and attention problems as well as GHQ-12), therefore following the recommendations by Howell, [35] we report multivariate tests (Pillai's Trace) for these five outcomes. The results show significant improvement over time for all outcomes (see Table 2 for means, standard deviations, mean differences, F values, and 95% confidence intervals [CIs] and effect sizes).

For parent-reported CBCL, repeated measure ANOVA revealed significant improvements in children for all primary outcomes from baseline to the three-year follow-up: total problem scores (95% CI, 11.49–18.00), internalizing problems (95% CI, 4.43–6.24), and externalizing problems (95% CI, 2.48–5.83). The effect sizes were all large. Similarly, significant improvements were observed for all secondary outcomes, i.e., CBCL scores for social,

For peer review only

thought, and attention problems in children, and parents' mental health as measured by GHQ-12. The associated effect sizes for the secondary outcomes were medium to large.

[Insert Table 2 here]

Clinical significance of change in children's and parents' mental health

Figure 2 shows the clinical significance of change in the mental health of children and parents at two-month and three-year follow-ups. The proportion of children who exhibited no clinically significant change from baseline to the two-month follow-up ranged from 61% on social problems to 88% on attention problems. This indicated that the level of various problems remained unchanged for most children. Very few children exhibited negative changes (4% to 8%), while positive changes were observed for a higher number of children (8% to 31%).

The positive changes were more pronounced from baseline to the three-year follow-up: 25% to 57% of children demonstrated clinically significant improvement on the primary outcomes, while 12% to 47% demonstrated clinically significant improvement on the secondary outcomes. No negative changes were observed for children in any of the outcomes.

Most parents showed no clinically significant changes from baseline to the two-month or three-year follow-ups (71% and 82%, respectively). Very few showed negative changes (6% and 2% at the two-month and three-year follow-ups, respectively), while positive changes were observed for a higher number of parents (20% and 12% at the two-month and three-year follow-ups, respectively).

[Insert Figure 2 here]

Supplementary and sensitivity analyses. Long-term follow-up data was available for 36 out of 39 parents in the control group who had received the intervention. One-way repeated measure

For peer review only

ANOVAs on this group revealed similar significant improvement from baseline to the long-term follow-up for all outcomes except CBCL attention problems, which had remained the same. In addition, we imputed the data for the six participants who had dropped out from the study using the worst available scores (i.e., worst case scenario). One-way repeated measure ANOVAs on the imputed data revealed similar significant improvement from baseline to the long-term follow-up for all outcomes. These results point to the robustness of the main findings.

For peer review only

DISCUSSION

To the best of our knowledge, this is the first study to evaluate the long-term effect of a culturally tailored parenting support program on the mental health of immigrant parents and their children. Our findings reveal that all the short-term positive changes in the mental health of children and parents were maintained three years post-intervention. The findings also suggest that clinically positive changes were generally retained over time.

Our findings are in line with previous systematic and meta-analytical reviews, [36, 37] which have indicated that positive change in children's emotional and behavioral problems is sustained over time. However, the parenting programs evaluated in these previous studies were mainly based on cognitive behavioral theories. In contrast, we tested the Connect program in our study, which is based on attachment theory. Our findings are also consistent with another Swedish study on non-immigrant parents that showed that the Connect program had continuously improved children's behavioral and emotional problems two years after the intervention [38], although the related effect sizes were generally larger in our study.

The improvement in children's mental health can be explained by parents' reflectivity and sensitivity towards their children's emotional needs, which is the aim of the Connect program. [39] Our previous qualitative study [29] revealed that after participating in the Connect program, parents became more aware of their children's expressed and non-expressed needs and made themselves more emotionally available to them. Parents also reported using the principles in the Connect program, such as 'taking a step back'. [29] The positive results can also be attributed to factors unrelated to the intervention. For example, as children grow older, teenage problems and parent-adolescent conflicts diminish, meaning parents report fewer problems.

For peer review only

In this study, a smaller change was detected in parents' mental health. Although parenting support programs do not aim to improve parents' mental health directly, studies have shown that parents can benefit from parenting support programs that are tailored to their needs. [26, 40-42] However, other external factors (such as employment, acculturation, perceived discrimination, and social isolation) might play a more important role in parental mental health. [7, 12, 13, 43, 44] Therefore, the benefits associated with participation in parenting programs might be more difficult to maintain over time.

Strengths and limitations

Several strengths and limitations of this study should be taken into consideration. First, there are few studies on the long-term effect of parenting programs, particularly those delivered to immigrant parents. [45] Second, most studies with long-term follow-ups have high-dropout rates. In our study, we were successful in retaining 85% of our original sample.

The main limitation of the study was the lack of a control group, which only allowed within-group analyses. The control group was offered the intervention shortly after the two-month follow up due to ethical and practical reason. The collaborator municipality was resolved that the intervention should be given to the parents in the control group because of its short-term positive impact on participating families.

Another limitation, similar to many other studies, was that intervention was evaluated using parental reports only, which may be biased. It is preferable to collect data from parents, children, and teachers. In our study, parents might have had reservations about reporting problems, as highlighted in previous studies. [7, 22] We found that parents reported a very low number of problems at the three-year follow-up. However, this can be normative for children at this age, or it might indicate that attending the program changed parental perceptions of their children's behavior. Including children's self-reports would have

For peer review only

strengthened the results. We had, in fact, initially intended to evaluate the intervention using both parents' and children's reports. However, the vast majority of the participating parents refused to consent to their children's direct participation in the study. This was mainly due to their (mis)perception of child protection policies in Sweden; they were afraid that the children might, accidently or intentionally, say something true or untrue that would lead to the involvement of Family and Child Welfare Services and their children would potentially be taken away. Interestingly, at the three-years follow-up, parents readily agreed with their children participation in a focus group discussion. This implies that addressing mis-conceptions about child protection policies and building a long-term relationship with immigrant communities might facilitate the issues regarding children's participation in research studies.

Conclusion and implication for clinical practice

Previous studies have shown that participation in a culturally tailored parenting program was associated with positive changes in the mental health of Somali-born parents and their children. In the current study, we showed that these positive changes were maintained three years post-intervention. Given the study's limitations, our findings need to be replicated. Future studies should strive to measure the long-term effects of parenting programs for immigrant parents using a control group, and by including ratings from parents, children and teachers. Despite the reported limitations, the study highlights the potential benefits of offering a culturally tailored parenting support program to immigrant families.

Acknowledgement

The authors would like to thank all the parents who participated in this study. We also thank research assistant Abdikerim Mohamed Sh. Hassan for his important work in the data collection, and the Kamprad Foundation for funding this study.

Competing Interests

The authors have no conflicts of interest relevant to this article to disclose.

Funding and all other required statements

This work was supported by the Kamprad Foundation, grant number 2017:0069.

Contributors' statement

All authors conceptualized and designed the study.

Dr Osman, Dr Vixner, and Dr Salari were responsible for data analyses and interpretation. They drafted the initial manuscript, reviewed, revised, and approved the final manuscript as submitted.

Dr Flacking reviewed the data analyses and interpretation. She revised, reviewed, and approved the final manuscript as submitted.

Dr Schön reviewed, revised, and approved the final manuscript as submitted.

Dr Klingberg-Allvin reviewed, revised, and approved the final manuscript as submitted.

All authors approved the final manuscript as submitted.

Data sharing statement

The data sets for this study is not open access, but can be made available upon request to the Principal Investigator (FO) and according to the ethical approval.

Patient and Public involvement statement

A reference group consisting of key persons from the Somali community, Somali associations, Social Services, School Health and other stakeholder organisations were engaged in our original study. The reference group has given feedback on the initial design, conducting and disseminating the research.

For peer review only

REFERENCES

1. Eurostat. Migration and migrant population statistics. https://ec.europa.eu/eurostat/statistics-explained/index.php/Migration_and_migrant_population_statistics#Migrant_population:_22.3_million_non-EU_citizens_living_in_the_EU_on_1_January_2018. Published 2019. Accessed 20 October, 2019.
2. Bhugra D. Migration and mental health. *Acta Psychiatri Scand.* 2004;109(4):243-258.
3. Gilliver SC, Sundquist J, Li X and Sundquist K. Recent research on the mental health of immigrants to Sweden: a literature review. *Eur J Public Health.* 2014;24 Suppl 1:72-79.
4. Kirmayer LJ, Narasiah L, Munoz M, et al. Common mental health problems in immigrants and refugees: general approach in primary care. *Can. Med. Assoc. J.* 2011;183(12):E959-E967.
5. Kouider EB, Koglin U and Petermann F. Emotional and Behavioral Problems in Migrant Children and Adolescents in American Countries: A Systematic Review. *J. Immigr. Minor Health.* 2015;17(4):1240-1258.
6. Nguyen H, Rawana JS and Flora DB. Risk and Protective Predictors of Trajectories of Depressive Symptoms Among Adolescents from Immigrant Backgrounds. *J. Youth Adolesc.* 2011;40(11):1544-1558.
7. Osman F, Klingberg-Allvin M, Flacking R and Schön U-K. Parenthood in transition – Somali-born parents' experiences of and needs for parenting support programmes. *BMC Int. Health Hum. Rights.* 2016;16(7):1-11.
8. Liebkind K and Jasinskaja-Lahti I. Acculturation and Psychological Well-Being among Immigrant Adolescents in Finland: A Comparative Study of Adolescents From Different Cultural Backgrounds. *J. Adolesc. Res.* 2000;15(4):446-469.
9. Lincoln AK, Lazarevic V, White MT and Ellis BH. The Impact of Acculturation Style and Acculturative Hassles on the Mental Health of Somali Adolescent Refugees. *J Immigr Minor Health.* 2015.
10. Lindencrona F, Ekblad S and Hauff E. Mental health of recently resettled refugees from the Middle East in Sweden: the impact of pre-resettlement trauma, resettlement stress and capacity to handle stress. *Soc. Psychiatry Psychiatr. Epidemiol.* 2008;43(2):121-131.
11. Miller KE and Rasmussen A. War exposure, daily stressors, and mental health in conflict and post-conflict settings: Bridging the divide between trauma-focused and psychosocial frameworks. *Social Science & Medicine.* 2010;70(1):7-16.
12. Warfa N, Bhui K, Craig T, et al. Post-migration geographical mobility, mental health and health service utilisation among Somali refugees in the UK: a qualitative study. *Health Place.* 2006;12(4):503-515.
13. Warfa N, Curtis S, Watters C, Carswell K, Ingleby D, Bhui K. Migration experiences, employment status and psychological distress among Somali immigrants: a mixed-method international study. *BMC Public Health.* 2012;12(1):1-12.
14. Dix T and Meunier LN. Depressive symptoms and parenting competence: An analysis of 13 regulatory processes. *Dev Rev.* 2009;29(1):45-68.
15. Gelkopf M and Jabotaro SE. Parenting style, competence, social network and attachment in mothers with mental illness. *Child Fam Soc Work.* 2013;18(4):496-503.
16. Haslam DM, Pakenham KI and Smith A. Social support and postpartum depressive symptomatology: The mediating role of maternal self-efficacy. *Infant Ment Health J.* 2006;27(3):276-291.

For peer review only

17. Park IJK, Kim PY, Cheung RYM and Kim M. The Role of Culture, Family Processes, and Anger Regulation in Korean American Adolescents' Adjustment Problems. *Am. J. Orthopsychiat.* 2010;80(2):258-266.
18. Stevens G, Vollebergh WAM, Pels TVM and Crijnen AAM. Parenting and internalizing and externalizing problems in Moroccan immigrant youth in the Netherlands. *J. Youth Adolesc.* 2007;36(5):685-695.
19. Bowie BH, Wojnar D and Isaak A. Somali Families' Experiences of Parenting in the United States. *West. J. Nur. Res.* 2017;39(2):273-289.
20. Lewig K, Arney F and Salveron M. Challenges to parenting in a new culture: Implications for child and family welfare. *Eval. Program Plann.* 2010;33(3):324-332.
21. Renzaho AMN, Green J, Mellor D and Swinburn B. Parenting, family functioning and lifestyle in a new culture: the case of African migrants in Melbourne, Victoria, Australia. *Child Fam Soc Work.* 2011;16(2):228-240.
22. Renzaho AMN, McCabe M and Sainsbury WJ. Parenting, role reversals and the preservation of cultural values among Arabic speaking migrant families in Melbourne, Australia. *Int. J. Intercult. Relat.* 2011;35(4):416-424.
23. Aronsson AP, Warner G and Sarkadi A, Osman F. "I'm a mother who always tries to give my children hope"- Refugee women's experiences of their children's mental health. *Front. Psychiatry.* 2019;10:789.
24. Bjorknes R and Manger T. Can Parent Training Alter Parent Practice and Reduce Conduct Problems in Ethnic Minority Children? A Randomized Controlled Trial. *Prev. Sci.* 2013;14(1):52-63.
25. Osman F, Flacking R, Schön U-K and Klingberg-Allvin M. A Support Program for Somali-born Parents on Children's Behavioral Problems. *Pediatrics.* 2017;139(3).
26. Osman F, Salari R, Klingberg-Allvin M, Schön U-K and Flacking R. Effects of a culturally tailored parenting support programme in Somali-born parents' mental health and sense of competence in parenting: a randomised controlled trial. *BMJ Open.* 2017;7(12).
27. Renzaho AM and Vignjevic S. The impact of a parenting intervention in Australia among migrants and refugees from Liberia, Sierra Leone, Congo, and Burundi: Results from the African Migrant Parenting Program. *J. Fam. Stud.* 2011;17(1):71-79.
28. Moretti MM, Braber K and Obsuth I. *Connect. An Attachment-focused treatment group for parents and caregivers–A principle based manual.* Burnaby, British Columbia, Canada: Simon Fraser University.; 2009.
29. Osman F, Flacking R, Klingberg Allvin M and Schön U-K. Qualitative study showed that a culturally tailored parenting programme improved the confidence and skills of Somali immigrants. *Acta Paediatr.* 2019;108(8):1482-1490.
30. Achenbach TM and Rescorla LA. *Manual for the ASEBA School-Age Forms & Profiles.* Burlington, VT: University of Vermont, Research Center for Children, Youth, & Families; 2001.
31. Goldberg DP, Gater R, Sartorius N, et al. The validity of two versions of the GHQ in the WHO study of mental illness in general health care. *Psychol. Med.* 1997;27:191-197.
32. World Health Organization W. Process of translation and adaptation of instruments. In: World Health Organazation; 2013.
33. Cohen J. A power primer. *Psychol. Bull.* 1992;112(1):155-159.
34. Jacobson NS and Truax P. Clinical significance: a statistical approach to defining meaningful change in psychotherapy research. *J. Consult. Clin. Psychol.* 1991;59(1):12.

For peer review only

35. Howell, D.C. (2009). *Statistical Methods for Psychology* (5th ed.). Pacific Grove CA: Duxbury.
36. Lundahl B, Risser H and Lovejoy M. A meta-analysis of parent training: moderators and follow-up effects. *Clin Psychol Rev.* 2006;26:86 - 104.
37. van Aar J, Leijten P, Orobio de Castro B and Overbeek G. Sustained, fade-out or sleeper effects? A systematic review and meta-analysis of parenting interventions for disruptive child behavior. *Clin. Psychol. Rev.* 2017;51:153-163.
38. Högström J, Olofsson V, Özdemir M, Enebrink P and Stattin H. Two-year findings from a national effectiveness trial: effectiveness of behavioral and non-behavioral parenting programs. *J. Abnorm. Child Psychol.* 2017;45(3):527-542.
39. Moretti MM and Obsuth I. Effectiveness of an attachment-focused manualized intervention for parents of teens at risk for aggressive behaviour: The Connect Program. *J. Adolesc.* 2009;32(6):1347-1357.
40. Griner D and Smith TB. Culturally adapted mental health interventions: A meta-analytic review. *Psychotherapy.* 2006;43(4):531-548.
41. Koerting J, Smith E, Knowles MM, et al. Barriers to, and facilitators of, parenting programmes for childhood behaviour problems: a qualitative synthesis of studies of parents' and professionals' perceptions. *Eur Child Adolesc Psych.* 2013;22(11):653-670.
42. Wells MB, Sarkadi A and Salari R. Mothers' and fathers' attendance in a community-based universally offered parenting program in Sweden. *Scand J Public Health.* 2015.
43. Grant J and Guerin PB. Applying Ecological Modeling to Parenting for Australian Refugee Families. *J. Transcult. Nurs.* 2014;25(4):325-333.
44. Missinne S and Bracke P. Depressive symptoms among immigrants and ethnic minorities: a population based study in 23 European countries. *Soc. Psychiatry Psychiat. Epidemiol.* 2012;47(1):97-109.
45. Özdemir M. How much do we know about the long-term effectiveness of parenting programmes? Advances, shortcomings, and future directions. *J. Child. Serv.* 2015;10(2):120-132.

For peer review only

Table 1. Characteristics of intervention participants at baseline for those who remained in the study (n = 51) and those who dropped out after two-month follow-up (n = 6)

	Remained		Dropped out	
**Categorical variables**	***n***	**%**	***n***	**%**
Parents				
Mothers	34	66.7	6	100.0
Fathers	17	33.3	0	0.0
Identified child's gender				
Girls	32	62.7	2	33.3
Boys	19	37.3	4	66.7
Years in Sweden [a]				
1-5 years	20	39.2	1	16.7
6 years or more	31	60.8	5	83.3
Highest educational level				
Less than upper secondary school	29	56.9	6	100.0
Upper secondary school or more	22	43.1	0	0.0
Occupation				
Employed	5	9.8	0	0.0
Other	46	90.2	6	100.0
Civic status				
Married	33	64.7	4	66.7
Single	18	35.3	2	33.3
Cohabiting with partner				
Yes	30	58.8	1	16.7
No	21	41.2	5	83.3
Worries about own financial situation [b]				
Yes	17	34.0	3	50.0

For peer review only

No		33	66.0		3	50.0	
**Continuous variables**		***M***	***SD***		***M***	***SD***	
Identified child's age at baseline		13.51	1.61		14.17	2.14	
Participants' age		43.80	7.77		46.33	11.08	
Number of children living at home [c]		5.74	2.77		4.33	2.94	

Note. No statistically significant differences were found between the two groups

[a] None of the parents in our sample had lived in any other Western country before arriving in Sweden. Typically, the journey from Somali to Sweden takes less than a year with some Somalis having to wait in Somali's neighboring countries before continuing their journey to Europe and resettling in Sweden; [b] n = 50 for those who remained in the study; [c] n = 49 for those who remained in the study

For peer review only

Table 2. Within-group comparison of intervention participants at baseline, two-month follow-up, and three-year follow-up (N = 51)

					Within-subject change							
					**Baseline to 2-month FU**				**Baseline to 3-year FU**			
	**Baseline Mean (SD)**	**2-month FU Mean (SD)**	**3-year FU Mean (SD)**	***F* (2, 49)** [a]	**Mean diff**	***F* (1, 50)** [b]	**95% CI**	***d***	**Mean diff**	***F* (1, 50)** [b]	**95% CI**	***d***
**CBCL 6-18**												
Total problem score	15.71 (9.8)	9.49 (7.4)	0.96 (2.1)	95.86***	6.22	15.13***	2.26-10.18	0.54	14.75	125.84***	11.49-18.00	1.57
Internalizing	5.65 (2.6)	3.76 (2.9)	0.31 (0.8)	84.08***	1.88	15.98***	0.72-3.05	0.56	5.33	212.50***	4.43-6.24	2.04
Externalizing	4.67 (5.2)	2.39 (3.5)	0.51 (1.4)	26.94***	2.28	8.28*	0.32-4.23	0.40	4.12	37.77***	2.48-5.83	0.86
Social problems	1.65 (1.5)	0.76 (1.2)	0.02 (0.1)	35.95***	0.88	12.78**	0.27-1.49	0.50	1.63	61.44***	1.11-2.14	1.10
Thought problems	0.61 (0.8)	0.53 (0.9)	0.08 (0.3)	9.83***	0.08	0.29	-0.28-0.44	0.08	0.53	23.27***	0.26-0.80	0.68
Attention problems	1.23 (2.2)	0.61 (1.3)	0.04 (0.2)	11.80***	0.63	3.72	-0.18-1.43	0.27	1.20	15.59***	0.45-1.95	0.55
**GHQ-12**[a]	20.00 (4.2)	17.80 (4.8)	18.22 (2.3)	5.46**	2.18	7.37*	0.19-4.18	0.41	1.76	10.28**	0.40-3.11	0.46

[a] $F(2, 47)$ for GHQ-12

[b] p values adjusted for multiple comparison (Bonferroni); $F(1, 48)$ for GHQ-12

CBCL= Child Behaviour Checklist, GHQ = General Health Questionnaire; FU = follow-up

Grey marked indicate primary outcomes

*** $p < .001$; ** $p < .01$; * $p < .05$

For peer review only

For peer review only - http://bmjopen.bmj.com/site/about/guidelines.xhtml

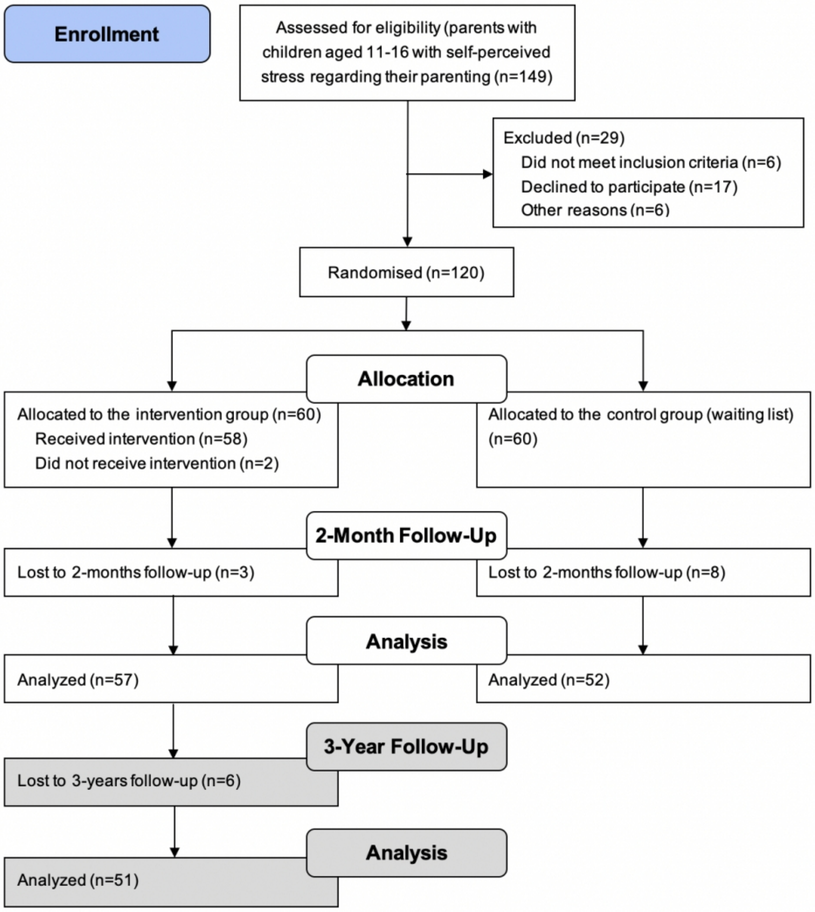

Figure 1. Participant flowchart throughout the study (grey marked area concerns the current study)

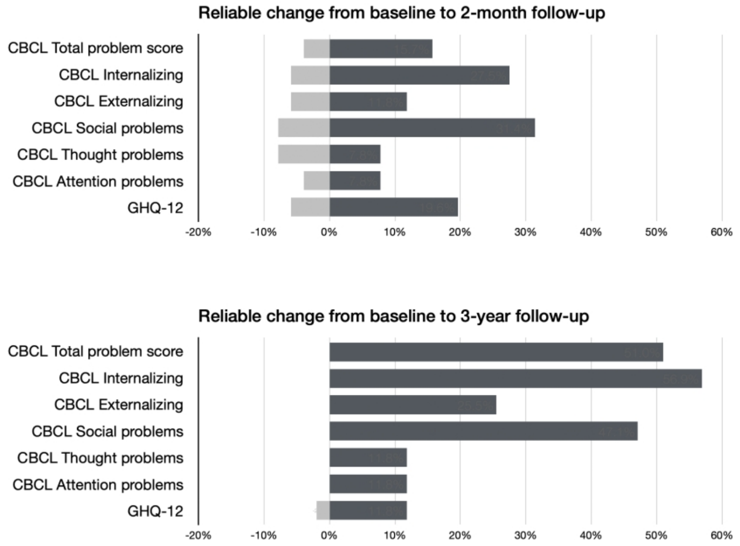

CBCL= Child Behaviour Checklist, GHQ = General Health Questionnaire

Reliable negative change marked in light grey and reliable positive change in dark grey

Figure 2. Clinical significance of change from baseline: proportion of children (CBCL) and parents (GHQ) showing reliable change (N =51 for children and 49 for parents)

BMJ Open

The impact of a culturally tailored parenting program on the mental health of Somali parents and children living in Sweden: A longitudinal cohort study

Journal:	*BMJ Open*
Manuscript ID	bmjopen-2020-045067.R1
Article Type:	Original research
Date Submitted by the Author:	22-Feb-2021
Complete List of Authors:	Osman, Fatumo; Hogskolan Dalarna, School of Education, Health and Social Studies; Uppsala University, Department of Public Health and Caring Sciences<br>Vixner, Linda; Hogskolan Dalarna, School of Education, Health and Social Studies<br>Flacking, Renee; Hogskolan Dalarna, School of Education, Health and Social Studies<br>klingberg-allvin, marie; Högskolan Dalarna, School of Education, Health and Social Studies<br>Schön, Ulla-Karin; Stockholm University, Department of Social Work<br>Salari, Raziye; Uppsala Universitet Institutionen for folkhalso- och vardvetenskap, Child Health and Parenting, Department of Public Health and Caring Sciences
**Primary Subject Heading**:	Mental health
Secondary Subject Heading:	Evidence based practice, Mental health, Public health, General practice / Family practice
Keywords:	MENTAL HEALTH, PUBLIC HEALTH, Child & adolescent psychiatry < PSYCHIATRY, Community child health < PAEDIATRICS

SCHOLARONE™
Manuscripts

I, the Submitting Author has the right to grant and does grant on behalf of all authors of the Work (as defined in the below author licence), an exclusive licence and/or a non-exclusive licence for contributions from authors who are: i) UK Crown employees; ii) where BMJ has agreed a CC-BY licence shall apply, and/or iii) in accordance with the terms applicable for US Federal Government officers or employees acting as part of their official duties; on a worldwide, perpetual, irrevocable, royalty-free basis to BMJ Publishing Group Ltd ("BMJ") its licensees and where the relevant Journal is co-owned by BMJ to the co-owners of the Journal, to publish the Work in this journal and any other BMJ products and to exploit all rights, as set out in our licence.

The Submitting Author accepts and understands that any supply made under these terms is made by BMJ to the Submitting Author unless you are acting as an employee on behalf of your employer or a postgraduate student of an affiliated institution which is paying any applicable article publishing charge ("APC") for Open Access articles. Where the Submitting Author wishes to make the Work available on an Open Access basis (and intends to pay the relevant APC), the terms of reuse of such Open Access shall be governed by a Creative Commons licence – details of these licences and which Creative Commons licence will apply to this Work are set out in our licence referred to above.

Other than as permitted in any relevant BMJ Author's Self Archiving Policies, I confirm this Work has not been accepted for publication elsewhere, is not being considered for publication elsewhere and does not duplicate material already published. I confirm all authors consent to publication of this Work and authorise the granting of this licence.

For peer review only

The impact of a culturally tailored parenting program on the mental health of Somali parents and children living in Sweden: A longitudinal cohort study

Fatumo Osman[a,c], Linda Vixner[a], Renée Flacking[a], Marie Klingberg-Allvin[a], Ulla-Karin Schön[b] & Raziye Salari[c]

[a] *School of Education, Health and Social Studies, Dalarna University, Falun, Sweden*

[b] *Department of Social Work, Stockholm University, Stockholm, Sweden*

[c] *Child Health and Parenting (CHAP), Department of Public Health and Caring Sciences, Uppsala University, Uppsala, Sweden*

Corresponding author: Fatumo Osman, Dalarna University, 79188 Falun, Sweden, Telephone work: +4623778220, Cell phone: +46701918604, Email: fos@du.se

For peer review only

ABSTRACT

Objectives: This study aimed to evaluate the long-term impact (three-year follow-up) of a culturally tailored parenting support program (Ladnaan) on the mental health of Somali-born parents and their children living in Sweden.

Methods: In this longitudinal cohort study, Somali-born parents with children aged 11–16 were followed up three years after they had participated in the Ladnaan intervention. The Ladnaan intervention comprises two main components: societal information, and the Connect parenting program delivered using a culturally sensitive approach. It consists of 12 weekly group-based sessions each lasting 1–2 hours. The primary outcome was improved mental health in children, as measured by the Child Behavior Checklist (CBCL). The secondary outcome was improved mental health in parents, as measured by the General Health Questionnaire (GHQ-12). Data were collected from the parent's perspective.

Results: Of the 60 parents who were originally offered the intervention, 51 were included in this long-term follow-up. The one-way repeated measures (baseline to the three-year follow-up) Analysis of Variance (ANOVA) for the CBCL confirmed maintenance of all the treatment gains for children: total problem scores (95% *CI*, 11.49–18.00, $d = 1.57$), internalizing problems (95% *CI*, 4.43–6.24, $d = 2.04$), and externalizing problems (95% CI, 2.48–5.83, $d = 0.86$). Similar results were observed for the parents' mental health (95% CI, 0.40–3.11, $d = 0.46$).

Conclusion: Positive changes in the mental health of Somali-born parents and their children were maintained three years after they had participated in a parenting support program that was culturally tailored and specifically designed to address their needs. Our findings highlight the long-term potential benefits of these programs in tackling mental health issues in immigrant families.

For peer review only

Clinical Trial: The original randomized controlled trial has been registered at www.clinicaltrials.gov (identifier NCT02114593). In the current study, we focus on the intervention group only.

ARTICLE SUMMARY

Strengths and limitations of the study

- The research used a longitudinal cohort study design (three-year follow-up) of an intervention group who received support from a culturally tailored parenting program.
- The majority of the original sample were retained in this three-year follow-up.
- The main limitation of the study was the lack of a control group, which only allowed within-group analyses.
- The control group was offered the intervention shortly after the two-month follow-up due to ethical and practical reasons.
- Data were collected through parental self-report.

For peer review only

INTRODUCTION

Over the past 20 years, Sweden and many other European countries have experienced a rapid increase in immigration, particularly immigrants who have fled from areas of war. [1] Stress factors due to immigration have a negative influence on the mental health of parents and their children. [2-7] More specifically, studies have shown that post-migration factors such as lack of social networks and support, perceived discrimination in the host county, inadequate accommodation, and acculturation challenges contribute to behavioral problems in children.. [7-13] Studies have also reported that the mental health problems of immigrant parents are associated with low confidence in parenting [14-16] and the use of authoritarian parenting, which has been shown to be related to children externalizing problems. [6, 17, 18]. In addition to negative parenting practices, power conflicts between parents and children are also associated with more behavioral problems among immigrant children. [17, 18].

There is a large body of evidence indicating that parenting support programs promote positive parenting practices and parent-child relationships; they also decrease children's internalizing and externalizing problems and improve parents' mental health problems. [19, 20] However, parents with an immigrant background have reported a need for culturally tailored parenting support programs that assist them in adapting their parenting to the host country, improving their relationships with their children, [7, 21-24] and improving their children's mental health. [25] Few studies have attempted to culturally tailor parenting programs to immigrant families. These studies have reported that culturally tailored parenting programs improve children's mental health, strengthen parenting roles, and enhance parent-child relationships in the short-term. [26-29] However, little is known about the long-term effects of these programs on the mental health of parents and their children.

For peer review only

Objectives

This study aimed to evaluate the long-term impact (three-year follow-up) of a culturally tailored parenting support program (Ladnaan) on the mental health of Somali-born parents and their children living in Sweden.

METHODS

Study design

This cohort study is a longitudinal (three-year) follow-up of the intervention group in a randomized controlled trial [27, 28] which evaluated the impact of a culturally tailored parenting support program for Somali-born parents living in Sweden, referred to as the Ladnaan intervention. Ethical approval was obtained from the Swedish Regional Ethical Review Board in Uppsala, Sweden (Dnr 2014/048/2), and the trial was registered at www.clinicaltrials.gov (identifier NCT02114593).

For peer review only

Originally, the study included a two-month and a six-month post-intervention follow-up for both intervention and control groups. However, the participating municipality started offering the intervention to the control group shortly after the two-month follow-up data collection. As we had lost most of our control group by the sixth month, we opted for a three-year post-intervention follow-up instead.

Setting and participants

The study was conducted in a town in the middle of Sweden with approximately 50,000 inhabitants, approximately 3,000 of whom are of Somali origin. Baseline (pre-intervention) and two-month follow-up data were collected between May 2014 and October 2015. The three-year post-intervention data were collected between April and October 2018. Data were

collected by the first author and by research assistants who were trained in the recruitment process, taking informed consent, and the use of the instruments employed in the study.

The main trial included 120 Somali-born parents with children aged 11–16 years and self-perceived stress related to parenting practices. If the parents had more than one child aged 11–16, they were asked to complete the questionnaire for the child with whom they particularly wished to improve their relationship. In two-parent families, both parents were offered the possibility of participating in the intervention, but, throughout the study, only the parent who was screened at baseline completed the questionnaire. Originally, we had also planned to collect data from the children. However, the parents did not consent to their children's direct participation in the study (mainly due to the fear of Social Services). Thus, only parent-reported data were available.

Baseline data were collected prior to randomization and the follow up data were collected two months as well as three years post-intervention. In comparison to the parents in the control group, the parents in the intervention group reported significant improvement in their children's and their own mental health at the two-month follow-up. [27, 28] Since most of the parents in the control group received the intervention soon after the two-month follow up, the present paper focuses on the three-year follow-up of the original intervention group. Of the 60 parents in the intervention group who completed the baseline assessment, three were lost to the two-month follow-up, and six were lost to the three-year follow-up. Thus, 51 parents in the intervention group were followed up three years post-intervention (see Figure 1).

[Insert Figure 1 here]

Patient and Public involvement statement

A reference group consisting of key persons from the Somali community, Somali associations, Social Services, School Health and other stakeholder organisations were

For peer review only

engaged in our original study. The reference group has given feedback on the initial design, conducting and disseminating the research.

Intervention

The Ladnaan intervention, developed based on a qualitative study conducted prior to the trial, [7] consists of two main components: societal information and the Connect parenting program. [30] It included 12 weekly sessions with groups of 12–17 parents, each lasting approximately 1–2 hours.

The first two sessions covered societal information relevant to Somali-born parents, including an overall view of the Swedish Child Welfare Services, the Convention on the Rights of the Child, and parenting styles in Sweden. The societal information component was developed through a qualitative study conducted with Somali-born parents that aimed to explore Somali-born parents' need for parenting support. [7] The other 10 sessions covered the Connect parenting support program, which is a standardized program based on attachment theory. The Connect program addresses nine different principles of child development, parent–child relationships, and challenging interactions, and included role playing, case examples, and reflection exercises. [30] (Detailed information of each session is shown in online supplemental table 1). The cultural sensitivity delivery approach was employed to ensure the sustainability of the parenting program after the study was finalized. This delivery approach incorporated elements, such as delivering the program in the participants' native language, using group leaders of similar background and experience, and culturally tailoring the role playing and case examples to make them understandable to and relatable for the participants. Only the parenting style theme in the societal information sessions was delivered by a Swedish-speaking professional from the Family and Child Welfare Services and interpreted by one of the group leaders.

For peer review only

In connection with every session, the parents were offered support for reading and writing letters from the authorities, such as the migration agency, social welfare services and hospitals.

Outcomes

The *primary outcomes* in this study were the Child Behavior Checklist (CBCL) scores for the total problems and internalizing and externalizing problems. The *secondary outcomes* were the CBCL scores for syndromes (social, thought, and attention problems) as well as the General Health Questionnaire (GHQ-12) scores.

The CBCL for children aged 6 to 18 years (CBCL 6–18) [30] was used to measure the children's mental health from the parents' perspective. We opted to use the CBCL 6–18 even for the children who had already turned 18 at the three-year follow-up because: (1) all the children were still living at home with their parents and (2) using the adult version would have led to difficulties in estimating the changes in the level of problems from baseline. The CBCL 6–18 consists of 133 items encompassing two sections: (1) competence scales (20 items on activities and social scales) and (2) emotional and behavioral problems (113 items). In this study, only items related to emotional and behavioral problems were used. According to the Achenbach System of Empirically Based Assessment (ASEBA) guideline [31], alcohol- and sex-related items can be excluded for Muslim respondents. In this study, five sex-related items were considered culturally inappropriate for our target population; thus, they were omitted, leaving 107 items in the CBCL. There were three possible response options for each problem item: 0 (not true), 1 (somewhat or sometimes true), and 2 (very true or often true). Using the ASEBA manual for CBCL 6–18 [31], we constructed six variables: a total problem score, two broad groupings of emotional and behavioral problems (internalizing and externalizing problems), and three syndrome scales (social, thought, and attention problems).

For peer review only

Higher scores indicate greater problem severity. The α Cronbach coefficients for the subscales in CBCL used in this study were > 0.78.

The 12-item version of the GHQ-12 [32] was used to measure the parents' overall mental health over the previous few weeks. Each item was rated on a four-point Likert scale from 1 (better than usual) to 4 (much worse than usual), with higher scores indicating higher mental health distress. The α Cronbach coefficients for GHQ-12 in this study were ≥ 0.80.

Both CBCL 6–18 and GHQ-12 were translated into Somali following the five steps of the World Health Organization's process of translation. [33] The translation started with forward translation (from English to Somali) by the first author and an interpreter who worked independently. After the translations were compared, a reconciled version was agreed upon. In the second step, backward translation was conducted by a bilingual social worker. In the third step, the backward translation was compared with the original version and then with the Somali version to check if the forward translation was accurate. In the fourth step, the Somali version of CBCL 6–18 and GHQ-12 were pilot tested with three mothers and fathers for accuracy. In the last step, the revision was proofread and approved by the ASEBA for CBCL 6–18 and by Mapi Research Trust for GHQ-12.

Statistical methods

All analyses were performed using IBM SPSS Statistics (Version 24). There were a few cases of missing data at the item level because some participants failed to answer all the questions. Thus, we started the analysis by reconstructing the mean outcome scores for participants who had at least filled in 70% of the items on each scale or subscale. This resulted in the retention of all 51 cases for CBCL and 49 cases for GHQ-12 in the analyses.

Descriptive statistics of baseline data were presented with mean (*SD*) or proportions (%). One-way repeated measure Analysis of Variances (ANOVAs) were used to compute the

For peer review only

within-subject change over time from baseline to two-month follow-up and three-year follow-up. Cohen's *d* effect size was calculated from *F* tests ($d = 0.2$, small; $d = 0.5$, medium; and $d = 0.8$, large). [34]

In addition, the clinical significance of change was measured from baseline to two-month follow-up and three-year follow-up using the method recommended by Jacobson and Truax. [35] Population norms for the CBCL 6–18 and GHQ-12 were not available for the present study population. Therefore, we used the pretest scores for all participants in the original randomized controlled trial to calculate the standard error of differences (S_{diff}). We assumed a measurement reliability of 0.8 for each measure. The clinical significance of change was then presented as the proportion of children/parents who had deteriorated, remained unchanged, or improved at two-month and three-year follow-ups.

For peer review only

RESULTS

Attrition and baseline characteristics

Table 1 presents baseline characteristics of the participants who remained in the study at the three-year follow-up and those who dropped out. No statistically significant differences were found between the two groups. Of the 51 participants who remained at the follow-up and were included in the analyses, 34 were mothers and 17 were fathers. Only five of them were employed at the time. Most of them had not entered upper secondary school, were married and living with their partner. The six participants who dropped out of the study at the three-year follow-up were all mothers, were educated up to upper secondary school level only, and were unemployed. Five of these six were not cohabiting with a partner.

A majority of the parents (n=40) attended at least 8 of the 12 intervention sessions. Most parents also received support from the group leaders in the form of reading letters from government agencies. However, this information was not formally recorded.

[Insert Table 1 here]

Long-term findings

Although most variables were not normally distributed, ANOVA is considered relatively robust to violations of normality assumptions [36, 37]

Mauchly's test indicated that the assumption of sphericity had been violated for five outcomes (CBCL total problem scores, externalizing, social problems and attention problems as well as GHQ-12), therefore following the recommendations by Howell, [38] we report multivariate tests (Pillai's Trace) for these five outcomes. The results show significant improvement over time for all outcomes (see Table 2 for means, standard deviations, mean differences, *F* values, and 95% confidence intervals [*CI*s] and effect sizes).

For peer review only

For parent-reported CBCL, repeated measure ANOVA revealed significant improvements in children for all primary outcomes from baseline to the three-year follow-up: total problem scores (95% *CI*, 11.49–18.00), internalizing problems (95% *CI*, 4.43–6.24), and externalizing problems (95% *CI*, 2.48–5.83). The effect sizes were all large. Similarly, significant improvements were observed for all secondary outcomes, i.e., CBCL scores for social, thought, and attention problems in children, and parents' mental health as measured by GHQ-12. The associated effect sizes for the secondary outcomes were medium to large.

[Insert Table 2 here]

Clinical significance of change in children's and parents' mental health

Figure 2 shows the clinical significance of change in the mental health of children and parents at two-month and three-year follow-ups. The proportion of children who exhibited no clinically significant change from baseline to the two-month follow-up ranged from 61% on social problems to 88% on attention problems. This indicated that the level of various problems remained unchanged for most children. Very few children exhibited negative changes (4% to 8%), while positive changes were observed for a higher number of children (8% to 31%).

The positive changes were more pronounced from baseline to the three-year follow-up: 25% to 57% of children demonstrated clinically significant improvement on the primary outcomes, while 12% to 47% demonstrated clinically significant improvement on the secondary outcomes. No negative changes were observed for children in any of the outcomes.

Most parents showed no clinically significant changes from baseline to the two-month or three-year follow-ups (71% and 82%, respectively). Very few showed negative changes (6% and 2% at the two-month and three-year follow-ups, respectively), while positive changes

For peer review only

were observed for a higher number of parents (20% and 12% at the two-month and three-year follow-ups, respectively).

[Insert Figure 2 here]

Supplementary and sensitivity analyses. Long-term follow-up data were available for 36 out of 39 parents in the control group who had received the intervention. One-way repeated measure ANOVAs on this group revealed similar significant improvement from baseline to the long-term follow-up for all the outcomes except CBCL attention problems, which had remained the same. In addition, we imputed the data for the six participants who had dropped out from the study using the worst available scores (i.e., worst case scenario). One-way repeated measure ANOVAs on the imputed data revealed similar significant improvement from baseline to the long-term follow-up for all the outcomes. These results point to the robustness of the main findings.

For peer review only

DISCUSSION

To the best of our knowledge, this is the first study to evaluate the long-term effect of a culturally tailored parenting support program on the mental health of immigrant parents and their children. Our findings reveal that all the short-term positive changes in the mental health of children and parents were maintained three years post-intervention. The findings also suggest that clinically positive changes were generally retained over time.

Our findings are in line with previous systematic and meta-analytical reviews, [39, 40] which have indicated that positive change in children's emotional and behavioral problems is sustained over time. However, the parenting programs evaluated in these previous studies were mainly based on cognitive behavioral theories. In contrast, we tested the Connect program in our study, which is based on attachment theory. Our findings are also consistent with another Swedish study on non-immigrant parents that showed that the Connect program had continuously improved children's behavioral and emotional problems two years after the intervention [41], although the related effect sizes were generally larger in our study.

The improvement in children's mental health can be explained by parents' reflectivity and sensitivity towards their children's emotional needs, which is the aim of the Connect program. [42] Our previous qualitative study [43] revealed that after participating in the Connect program, parents became more aware of their children's expressed and non-expressed needs and made themselves more emotionally available to them. Parents also reported using the principles in the Connect program, such as 'taking a step back'. [43] The positive results can also be attributed to factors unrelated to the intervention. For example, as children grow older, teenage problems and parent-adolescent conflicts diminish, meaning parents report fewer problems.

For peer review only

In this study, a smaller change was detected in parents' mental health. Although parenting support programs do not aim to improve parents' mental health directly, studies have shown that parents can benefit from parenting support programs that are tailored to their needs. [44-46] However, other external factors (such as employment, acculturation, perceived discrimination, and social isolation) might play a more important role in parental mental health. [7, 12, 13, 47, 48] Therefore, the benefits associated with participation in parenting programs might be more difficult to maintain over time.

Strengths and limitations

Several strengths and limitations of this study should be taken into consideration. First, there are few studies on the long-term effect of parenting programs, particularly those delivered to immigrant parents. [49] Second, most studies with long-term follow-ups have high-dropout rates. In our study, we were successful in retaining 85% of our original sample.

The main limitation of the study was the lack of a control group, which only allowed within-group analyses. The control group was offered the intervention shortly after the two-month follow up due to ethical and practical reason. The collaborator municipality was resolved that the intervention should be given to the parents in the control group because of its short-term positive impact on participating families.

Another limitation is that the outcome measures used in this study were not previously tested for their reliability and validity on this specific population. However, both measures are well-established questionnaires that have been readily used in cross-cultural settings showing good reliability and validity [32, 50].

Finally, similar to many other studies, the intervention was evaluated using parental reports only, which may be biased. It is preferable to collect data from parents, children, and teachers. In our study, parents might have had reservations about reporting problems, as highlighted in

For peer review only

previous studies. [7, 22] We found that parents reported a very low number of problems at the three-year follow-up. However, this can be normative for children at this age, or it might indicate that attending the program changed parental perceptions of their children's behavior. Including children's self-reports would have strengthened the results. We had, in fact, initially intended to evaluate the intervention using both parents' and children's reports. However, the vast majority of the participating parents refused to consent to their children's direct participation in the study. This was mainly due to their (mis)perception of child protection policies in Sweden; they were afraid that the children might, accidently or intentionally, say something true or untrue that would lead to the involvement of Family and Child Welfare Services and their children would potentially be taken away. Interestingly, at the three-years follow-up, parents readily agreed with their children participation in a focus group discussion. This implies that addressing mis-conceptions about child protection policies and building a long-term relationship with immigrant communities might facilitate the issues regarding children's participation in research studies.

Conclusion and implication for clinical practice

Previous studies have shown that participation in a culturally tailored parenting program was associated with positive changes in the mental health of Somali-born parents and their children. In the current study, we showed that these positive changes were maintained three years post-intervention. Given the study's limitations, our findings need to be replicated. Future studies should strive to measure the long-term effects of parenting programs for immigrant parents using a control group, and by including ratings from parents, children and teachers. Despite the reported limitations, the study highlights the potential benefits of offering a culturally tailored parenting support program to immigrant families.

For peer review only

Acknowledgement

The authors would like to thank all the parents who participated in this study. We also thank research assistant Abdikerim Mohamed for his important work in the data collection, and the Kamprad Foundation for funding this study.

Competing Interests

The authors have no conflicts of interest relevant to this article to disclose.

Funding and all other required statements

This work was supported by the Kamprad Foundation, grant number 2017:0069.

Contributors' statement

All authors conceptualized and designed the study.

Dr Osman, Dr Vixner, and Dr Salari were responsible for data analyses and interpretation. They drafted the initial manuscript, reviewed, revised, and approved the final manuscript as submitted.

Dr Flacking reviewed the data analyses and interpretation. She revised, reviewed, and approved the final manuscript as submitted.

Dr Schön reviewed, revised, and approved the final manuscript as submitted.

Dr Klingberg-Allvin reviewed, revised, and approved the final manuscript as submitted.

All authors approved the final manuscript as submitted.

Data sharing statement

The data sets for this study is not open access, but can be made available upon request to the Principal Investigator (FO) and according to the ethical approval.

For peer review only

REFERENCES

1. Eurostat. Migration and migrant population statistics. https://ec.europa.eu/eurostat/statistics-explained/index.php/Migration_and_migrant_population_statistics#Migrant_population:_22.3_million_non-EU_citizens_living_in_the_EU_on_1_January_2018. Published 2019. Accessed 20 October, 2019.
2. Bhugra D. Migration and mental health. *Acta Psychiatri Scand.* 2004;109(4):243-258.
3. Gilliver SC, Sundquist J, Li X and Sundquist K. Recent research on the mental health of immigrants to Sweden: a literature review. *Eur J Public Health.* 2014;24 Suppl 1:72-79.
4. Kirmayer LJ, Narasiah L, Munoz M, et al. Common mental health problems in immigrants and refugees: general approach in primary care. *Can. Med. Assoc. J.* 2011;183(12):E959-E967.
5. Kouider EB, Koglin U and Petermann F. Emotional and Behavioral Problems in Migrant Children and Adolescents in American Countries: A Systematic Review. *J. Immigr. Minor Health.* 2015;17(4):1240-1258.
6. Nguyen H, Rawana JS and Flora DB. Risk and Protective Predictors of Trajectories of Depressive Symptoms Among Adolescents from Immigrant Backgrounds. *J. Youth Adolesc.* 2011;40(11):1544-1558.
7. Osman F, Klingberg-Allvin M, Flacking R and Schön U-K. Parenthood in transition – Somali-born parents' experiences of and needs for parenting support programmes. *BMC Int. Health Hum. Rights.* 2016;16(7):1-11.
8. Liebkind K and Jasinskaja-Lahti I. Acculturation and Psychological Well-Being among Immigrant Adolescents in Finland: A Comparative Study of Adolescents From Different Cultural Backgrounds. *J. Adolesc. Res.* 2000;15(4):446-469.
9. Lincoln AK, Lazarevic V, White MT and Ellis BH. The Impact of Acculturation Style and Acculturative Hassles on the Mental Health of Somali Adolescent Refugees. *J Immigr Minor Health.* 2015.
10. Lindencrona F, Ekblad S and Hauff E. Mental health of recently resettled refugees from the Middle East in Sweden: the impact of pre-resettlement trauma, resettlement stress and capacity to handle stress. *Soc. Psychiatry Psychiatr. Epidemiol.* 2008;43(2):121-131.
11. Miller KE and Rasmussen A. War exposure, daily stressors, and mental health in conflict and post-conflict settings: Bridging the divide between trauma-focused and psychosocial frameworks. *Social Science & Medicine.* 2010;70(1):7-16.
12. Warfa N, Bhui K, Craig T, et al. Post-migration geographical mobility, mental health and health service utilisation among Somali refugees in the UK: a qualitative study. *Health Place.* 2006;12(4):503-515.
13. Warfa N, Curtis S, Watters C, Carswell K, Ingleby D, Bhui K. Migration experiences, employment status and psychological distress among Somali immigrants: a mixed-method international study. *BMC Public Health.* 2012;12(1):1-12.
14. Dix T and Meunier LN. Depressive symptoms and parenting competence: An analysis of 13 regulatory processes. *Dev Rev.* 2009;29(1):45-68.
15. Gelkopf M and Jabotaro SE. Parenting style, competence, social network and attachment in mothers with mental illness. *Child Fam Soc Work.* 2013;18(4):496-503.
16. Haslam DM, Pakenham KI and Smith A. Social support and postpartum depressive symptomatology: The mediating role of maternal self-efficacy. *Infant Ment Health J.* 2006;27(3):276-291.

For peer review only

17. Park IJK, Kim PY, Cheung RYM and Kim M. The Role of Culture, Family Processes, and Anger Regulation in Korean American Adolescents' Adjustment Problems. *Am. J. Orthopsychiat.* 2010;80(2):258-266.
18. Stevens G, Vollebergh WAM, Pels TVM and Crijnen AAM. Parenting and internalizing and externalizing problems in Moroccan immigrant youth in the Netherlands. *J. Youth Adolesc.* 2007;36(5):685-695.
19. Stewart-Brown, S., et al., Impact of a general practice based group parenting programme: quantitative and qualitative results from a controlled trial at 12 months. *Arch Dis Child* 2004;89(6):519-525.
20. Morgan, Z., et al., The effects of parent-child relationships on later life mental health status in two national birth cohorts. *Soc Psychiatry Psychiatr Epidemiol* 2012;47(11):1707-1715.
21. Bowie BH, Wojnar D and Isaak A. Somali Families' Experiences of Parenting in the United States. *West. J. Nur. Res.* 2017;39(2):273-289.
22. Lewig K, Arney F and Salveron M. Challenges to parenting in a new culture: Implications for child and family welfare. *Eval. Program Plann.* 2010;33(3):324-332.
23. Renzaho AMN, Green J, Mellor D and Swinburn B. Parenting, family functioning and lifestyle in a new culture: the case of African migrants in Melbourne, Victoria, Australia. *Child Fam Soc Work.* 2011;16(2):228-240.
24. Renzaho AMN, McCabe M and Sainsbury WJ. Parenting, role reversals and the preservation of cultural values among Arabic speaking migrant families in Melbourne, Australia. *Int. J. Intercult. Relat.* 2011;35(4):416-424.
25. Aronsson AP, Warner G and Sarkadi A, Osman F. "I'm a mother who always tries to give my children hope"- Refugee women's experiences of their children's mental health. *Front. Psychiatry.* 2019;10:789.
26. Bjorknes R and Manger T. Can Parent Training Alter Parent Practice and Reduce Conduct Problems in Ethnic Minority Children? A Randomized Controlled Trial. *Prev. Sci.* 2013;14(1):52-63.
27. Osman F, Flacking R, Schön U-K and Klingberg-Allvin M. A Support Program for Somali-born Parents on Children's Behavioral Problems. *Pediatrics.* 2017;139(3).
28. Osman F, Salari R, Klingberg-Allvin M, Schön U-K and Flacking R. Effects of a culturally tailored parenting support programme in Somali-born parents' mental health and sense of competence in parenting: a randomised controlled trial. *BMJ Open.* 2017;7(12).
29. Renzaho AM and Vignjevic S. The impact of a parenting intervention in Australia among migrants and refugees from Liberia, Sierra Leone, Congo, and Burundi: Results from the African Migrant Parenting Program. *J. Fam. Stud.* 2011;17(1):71-79.
30. Moretti MM, Braber K and Obsuth I. *Connect. An Attachment-focused treatment group for parents and caregivers–A principle based manual.* Burnaby, British Columbia, Canada: Simon Fraser University.; 2009.
31. Achenbach TM and Rescorla LA. *Manual for the ASEBA School-Age Forms & Profiles.* Burlington, VT: University of Vermont, Research Center for Children, Youth, & Families; 2001.
32. Goldberg DP, Gater R, Sartorius N, et al. The validity of two versions of the GHQ in the WHO study of mental illness in general health care. *Psychol. Med.* 1997;27:191-197.
33. World Health Organization W. Process of translation and adaptation of instruments. In: World Health Organazation; 2013.
34. Cohen J. A power primer. *Psychol. Bull.* 1992;112(1):155-159.

For peer review only

35. Jacobson NS and Truax P. Clinical significance: a statistical approach to defining meaningful change in psychotherapy research. *J. Consult. Clin. Psychol.* 1991;59(1):12.
36. Harwell M.R., et al., Summarizing Monte Carlo Results in Methodological Research: The One- and Two-Factor Fixed Effects ANOVA Cases. J. Educ. Stat. 1992. 17(4): 315-339.
37. Lix, L.M., J.C. Keselman, and H.J. Keselman, Consequences of Assumption Violations Revisited: A Quantitative Review of Alternatives to the One-Way Analysis of Variance "F" Test. Rev. Educ. Res. 1996. 66(4): 579-619.
38. Howell, D.C. (2009). *Statistical Methods for Psychology* (5th ed.). Pacific Grove CA: Duxbury.
39. Lundahl B, Risser H and Lovejoy M. A meta-analysis of parent training: moderators and follow-up effects. *Clin Psychol Rev.* 2006;26:86 - 104.
40. van Aar J, Leijten P, Orobio de Castro B and Overbeek G. Sustained, fade-out or sleeper effects? A systematic review and meta-analysis of parenting interventions for disruptive child behavior. *Clin. Psychol. Rev.* 2017;51:153-163.
41. Högström J, Olofsson V, Özdemir M, Enebrink P and Stattin H. Two-year findings from a national effectiveness trial: effectiveness of behavioral and non-behavioral parenting programs. *J. Abnorm. Child Psychol.* 2017;45(3):527-542.
42. Moretti MM and Obsuth I. Effectiveness of an attachment-focused manualized intervention for parents of teens at risk for aggressive behaviour: The Connect Program. *J. Adolesc.* 2009;32(6):1347-1357.
43. Osman F, Flacking R, Klingberg Allvin M and Schön U-K. Qualitative study showed that a culturally tailored parenting programme improved the confidence and skills of Somali immigrants. *Acta Paediatr.* 2019;108(8):1482-1490.
44. Griner D and Smith TB. Culturally adapted mental health interventions: A meta-analytic review. *Psychotherapy.* 2006;43(4):531-548.
45. Koerting J, Smith E, Knowles MM, et al. Barriers to, and facilitators of, parenting programmes for childhood behaviour problems: a qualitative synthesis of studies of parents' and professionals' perceptions. *Eur Child Adolesc Psych.* 2013;22(11):653-670.
46. Wells MB, Sarkadi A and Salari R. Mothers' and fathers' attendance in a community-based universally offered parenting program in Sweden. *Scand J Public Health.* 2015.
47. Grant J and Guerin PB. Applying Ecological Modeling to Parenting for Australian Refugee Families. *J. Transcult. Nurs.* 2014;25(4):325-333.
48. Missinne S and Bracke P. Depressive symptoms among immigrants and ethnic minorities: a population based study in 23 European countries. *Soc. Psychiatry Psychiat. Epidemiol.* 2012;47(1):97-109.
49. Özdemir M. How much do we know about the long-term effectiveness of parenting programmes? Advances, shortcomings, and future directions. *J. Child. Serv.* 2015;10(2):120-132.
50. Dumenci, L., et al., *Measurement Structure of the Turkish Translation of the Child Behavior Checklist Using Confirmatory Factor Analytic Approaches to Validation of Syndromal Constructs.* J. Abnorm Child Psychol, 2004. 32(3): p. 335-340.

For peer review only

Table 1. Characteristics of intervention participants at baseline for those who remained in the study (n = 51) and those who dropped out after two-month follow-up (n = 6)

	Remained		Dropped out	
**Categorical variables**	***n***	***%***	***n***	***%***
Parents				
Mothers	34	66.7	6	100.0
Fathers	17	33.3	0	0.0
Identified child's gender				
Girls	32	62.7	2	33.3
Boys	19	37.3	4	66.7
Years in Sweden [a]				
1-5 years	20	39.2	1	16.7
6 years or more	31	60.8	5	83.3
Highest educational level				
Less than upper secondary school	29	56.9	6	100.0
Upper secondary school or more	22	43.1	0	0.0
Occupation				
Employed	5	9.8	0	0.0
Other	46	90.2	6	100.0
Civic status				
Married	33	64.7	4	66.7
Single	18	35.3	2	33.3
Cohabiting with partner				
Yes	30	58.8	1	16.7
No	21	41.2	5	83.3
Worries about own financial situation [b]				
Yes	17	34.0	3	50.0

For peer review only

No		33	66.0		3	50.0	
**Continuous variables**		***M***	***SD***		***M***	***SD***	
Identified child's age at baseline		13.51	1.61		14.17	2.14	
Participants' age		43.80	7.77		46.33	11.08	
Number of children living at home [c]		5.74	2.77		4.33	2.94	

Note. No statistically significant differences were found between the two groups

[a] None of the parents in our sample had lived in any other Western country before arriving in Sweden. Typically, the journey from Somali to Sweden takes less than a year with some Somalis having to wait in Somali's neighboring countries before continuing their journey to Europe and resettling in Sweden; [b] n = 50 for those who remained in the study; [c] n = 49 for those who remained in the study

For peer review only

Table 2. Within-group comparison of intervention participants at baseline, two-month follow-up, and three-year follow-up (N = 51)

	Baseline	2-month FU	3-year FU		Within-subject change							
					Baseline to 2-month FU				Baseline to 3-year FU			
	Mean (*SD*)	Mean (*SD*)	Mean (*SD*)	F (2, 49) [a]	Mean diff	F (1, 50) [b]	95% *CI*	*d*	Mean diff	F (1, 50) [b]	95% *CI*	*d*
**CBCL 6-18**												
Total problem score	15.71 (9.8)	9.49 (7.4)	0.96 (2.1)	95.86***	6.22	15.13***	2.26-10.18	0.54	14.75	125.84***	11.49-18.00	1.57
Internalizing	5.65 (2.6)	3.76 (2.9)	0.31 (0.8)	84.08***	1.88	15.98***	0.72-3.05	0.56	5.33	212.50***	4.43-6.24	2.04
Externalizing	4.67 (5.2)	2.39 (3.5)	0.51 (1.4)	26.94***	2.28	8.28*	0.32-4.23	0.40	4.12	37.77***	2.48-5.83	0.86
Social problems	1.65 (1.5)	0.76 (1.2)	0.02 (0.1)	35.95***	0.88	12.78**	0.27-1.49	0.50	1.63	61.44***	1.11-2.14	1.10
Thought problems	0.61 (0.8)	0.53 (0.9)	0.08 (0.3)	9.83***	0.08	0.29	-0.28-0.44	0.08	0.53	23.27***	0.26-0.80	0.68
Attention problems	1.23 (2.2)	0.61 (1.3)	0.04 (0.2)	11.80***	0.63	3.72	-0.18-1.43	0.27	1.20	15.59***	0.45-1.95	0.55
**GHQ-12**[a]	20.00 (4.2)	17.80 (4.8)	18.22 (2.3)	5.46**	2.18	7.37*	0.19-4.18	0.41	1.76	10.28**	0.40-3.11	0.46

[a] $F(2, 47)$ for GHQ-12

[b] p values adjusted for multiple comparison (Bonferroni); $F(1, 48)$ for GHQ-12

CBCL= Child Behaviour Checklist, GHQ = General Health Questionnaire; FU = follow-up

Grey marked indicate primary outcomes

*** $p < .001$; ** $p < .01$; * $p < .05$

For peer review only

Figure 1: Participant flowchart throughout the study (grey marked area concerns the current study)

Figure 2. Clinical significance of change from baseline: proportion of children (CBCL) and parents (GHQ) showing reliable change (N= 51for children and 49 parents)

For peer review only

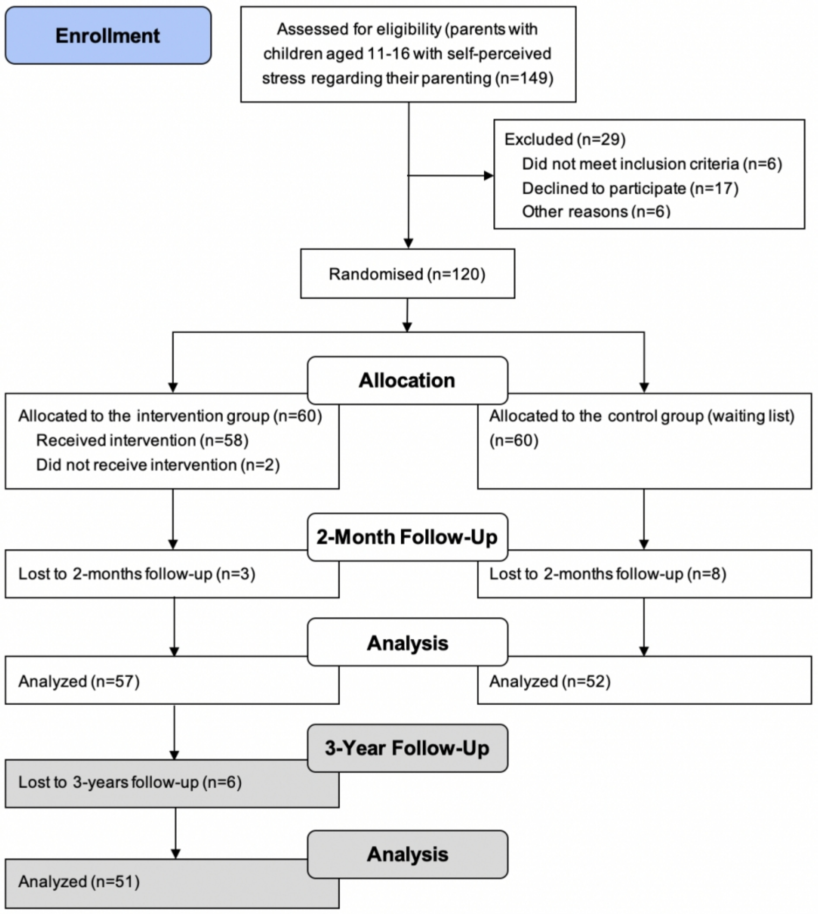

Figure 1. Participant flowchart throughout the study (grey marked area concerns the current study)

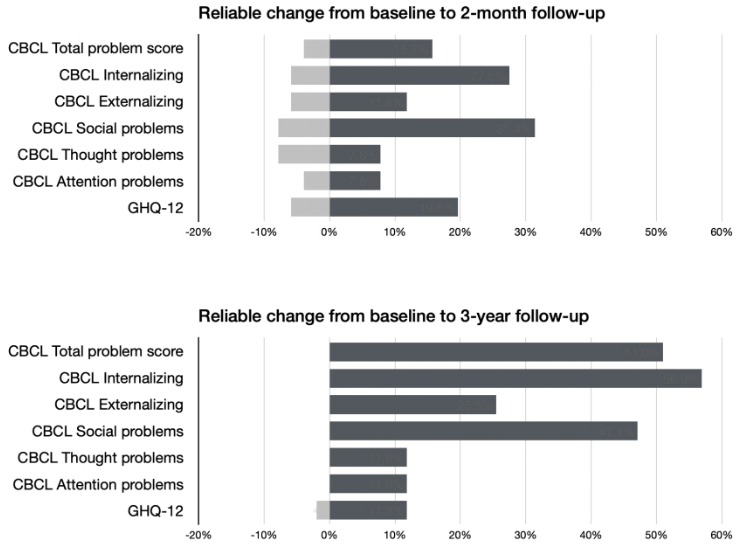

CBCL= Child Behaviour Checklist, GHQ = General Health Questionnaire

Reliable negative change marked in light grey and reliable positive change in dark grey

Figure 2. Clinical significance of change from baseline: proportion of children (CBCL) and parents (GHQ) showing reliable change (N =51 for children and 49 for parents)

Supplementary material

Table 1: Content of the intervention: Connect program and societal information

Session	Intervention topics	Content of the session
**Societal information**		
1	Child Welfare Services	- Knowledge and understanding on social service's work with children and youngsters.<br>- Various laws in the social services regarding the removal of the child from his/her family<br>- The support that social services have to offer for families
1	Parenting styles; authoritarian and democratic parenting	- Different parenting styles and the impact it has on child's developmental<br>- Knowledge and understanding of parental responsibility regarding personal, economic and social growth<br>- Interaction between children and parents in the new context
2	Convention on the rights of the child	- Knowledge on the United Nations Convention on the Rights of the Child<br>- Reflection on how parents follow the child's right<br>- Reflection on having a child perspective
- Connect program		
3	Introduction on Connect parenting program	- Introduction to Connect and the theory that the program is based on
4	All behaviour has a meaning	Children's behaviour and what it stands for, and how parents could recognize it
5	Attachment is for life	Skills to recognize the attachment needs of the infant, small children and teens
6	Conflict is a part of attachment	Conflict as a natural part of the relationship between parents and teens
7	Autonomous includes connection	The teens need regarding the autonomous while they want to be connected with their parents.
8	Empathy – the heartbeat of attachment	Practice and acknowledge empathy which is about being there for the child without condemning or giving solution of the problem.

For peer review only

Supplementary material

9	Balance our needs with needs of others	Parents to recognize their own attachment needs and look for other sources of support than their children.
10	Growth and change are part of relationship	Parents to become aware of their past, and acknowledge what can promote and hinder the teens change and growth.
11	Celebrating attachment	Acknowledgement of that in every relationship there are both joy and pain which should be celebrated.
12	Two steps forward, One step back: staying on course	Understand on how to respond when the relationship is on turbulence and how doing that can establish a reconciliation and strengthen the relationship.

For peer review only

STROBE 2007 (v4) Statement—Checklist of items that should be included in reports of *cohort studies*

Section/Topic	Item #	Recommendation	Reported on page #
**Title and abstract**	1	(*a*) Indicate the study's design with a commonly used term in the title or the abstract	1
		(*b*) Provide in the abstract an informative and balanced summary of what was done and what was found	2
**Introduction**			
Background/rationale	2	Explain the scientific background and rationale for the investigation being reported	4
Objectives	3	State specific objectives, including any prespecified hypotheses	5
**Methods**			
Study design	4	Present key elements of study design early in the paper	5
Setting	5	Describe the setting, locations, and relevant dates, including periods of recruitment, exposure, follow-up, and data collection	5-6
Participants	6	(*a*) Give the eligibility criteria, and the sources and methods of selection of participants. Describe methods of follow-up	5-6
		(*b*) For matched studies, give matching criteria and number of exposed and unexposed	n/a
Variables	7	Clearly define all outcomes, exposures, predictors, potential confounders, and effect modifiers. Give diagnostic criteria, if applicable	7-9
Data sources/ measurement	8*	For each variable of interest, give sources of data and details of methods of assessment (measurement). Describe comparability of assessment methods if there is more than one group	7-9
Bias	9	Describe any efforts to address potential sources of bias	9
Study size	10	Explain how the study size was arrived at	6
Quantitative variables	11	Explain how quantitative variables were handled in the analyses. If applicable, describe which groupings were chosen and why	9-10
Statistical methods	12	(*a*) Describe all statistical methods, including those used to control for confounding	9-10
		(*b*) Describe any methods used to examine subgroups and interactions	9-10
		(*c*) Explain how missing data were addressed	9
		(*d*) If applicable, explain how loss to follow-up was addressed	11-12
		(*e*) Describe any sensitivity analyses	13
**Results**			

For peer review only

Participants	13*	(a) Report numbers of individuals at each stage of study—eg numbers potentially eligible, examined for eligibility, confirmed eligible, included in the study, completing follow-up, and analysed	11
		(b) Give reasons for non-participation at each stage	11
		(c) Consider use of a flow diagram	Figure 1
Descriptive data	14*	(a) Give characteristics of study participants (eg demographic, clinical, social) and information on exposures and potential confounders	11
		(b) Indicate number of participants with missing data for each variable of interest	11
		(c) Summarise follow-up time (eg, average and total amount)	11
Outcome data	15*	Report numbers of outcome events or summary measures over time	11-12
Main results	16	(*a*) Give unadjusted estimates and, if applicable, confounder-adjusted estimates and their precision (eg, 95% confidence interval). Make clear which confounders were adjusted for and why they were included	11-12
		(*b*) Report category boundaries when continuous variables were categorized	11-12
		(*c*) If relevant, consider translating estimates of relative risk into absolute risk for a meaningful time period	n/a
Other analyses	17	Report other analyses done—eg analyses of subgroups and interactions, and sensitivity analyses	12-13
**Discussion**			
Key results	18	Summarise key results with reference to study objectives	14
**Limitations**			
Interpretation	20	Give a cautious overall interpretation of results considering objectives, limitations, multiplicity of analyses, results from similar studies, and other relevant evidence	14-16
Generalisability	21	Discuss the generalisability (external validity) of the study results	15-16
**Other information**			
Funding	22	Give the source of funding and the role of the funders for the present study and, if applicable, for the original study on which the present article is based	17

*Give information separately for cases and controls in case-control studies and, if applicable, for exposed and unexposed groups in cohort and cross-sectional studies.

Note: An Explanation and Elaboration article discusses each checklist item and gives methodological background and published examples of transparent reporting. The STROBE checklist is best used in conjunction with this article (freely available on the Web sites of PLoS Medicine at http://www.plosmedicine.org/, Annals of Internal Medicine at http://www.annals.org/, and Epidemiology at http://www.epidem.com/). Information on the STROBE Initiative is available at www.strobe-statement.org.

For peer review only

BMJ Open

The impact of a culturally tailored parenting program on the mental health of Somali parents and children living in Sweden: A longitudinal cohort study

Journal:	*BMJ Open*
Manuscript ID	bmjopen-2020-045067.R2
Article Type:	Original research
Date Submitted by the Author:	22-Jun-2021
Complete List of Authors:	Osman, Fatumo; Hogskolan Dalarna, School of Health and Welfare; Uppsala University, Department of Public Health and Caring Sciences<br>Vixner, Linda; Hogskolan Dalarna, School of Health and Welfare<br>Flacking, Renee; Hogskolan Dalarna, School of Health and Welfare<br>klingberg-allvin, marie; Högskolan Dalarna, School of Health and Welfare<br>Schön, Ulla-Karin; Stockholm University, Department of Social Work<br>Salari, Raziye; Uppsala Universitet Institutionen for folkhalso- och vardvetenskap, Child Health and Parenting, Department of Public Health and Caring Sciences
**Primary Subject Heading**:	Mental health
Secondary Subject Heading:	Evidence based practice, Mental health, Public health, General practice / Family practice
Keywords:	MENTAL HEALTH, PUBLIC HEALTH, Child & adolescent psychiatry < PSYCHIATRY, Community child health < PAEDIATRICS

SCHOLARONE™
Manuscripts

I, the Submitting Author has the right to grant and does grant on behalf of all authors of the Work (as defined in the below author licence), an exclusive licence and/or a non-exclusive licence for contributions from authors who are: i) UK Crown employees; ii) where BMJ has agreed a CC-BY licence shall apply, and/or iii) in accordance with the terms applicable for US Federal Government officers or employees acting as part of their official duties; on a worldwide, perpetual, irrevocable, royalty-free basis to BMJ Publishing Group Ltd ("BMJ") its licensees and where the relevant Journal is co-owned by BMJ to the co-owners of the Journal, to publish the Work in this journal and any other BMJ products and to exploit all rights, as set out in our licence.

The Submitting Author accepts and understands that any supply made under these terms is made by BMJ to the Submitting Author unless you are acting as an employee on behalf of your employer or a postgraduate student of an affiliated institution which is paying any applicable article publishing charge ("APC") for Open Access articles. Where the Submitting Author wishes to make the Work available on an Open Access basis (and intends to pay the relevant APC), the terms of reuse of such Open Access shall be governed by a Creative Commons licence – details of these licences and which Creative Commons licence will apply to this Work are set out in our licence referred to above.

Other than as permitted in any relevant BMJ Author's Self Archiving Policies, I confirm this Work has not been accepted for publication elsewhere, is not being considered for publication elsewhere and does not duplicate material already published. I confirm all authors consent to publication of this Work and authorise the granting of this licence.

For peer review only

The impact of a culturally tailored parenting program on the mental health of Somali parents and children living in Sweden: A longitudinal cohort study

Fatumo Osman[a,c], Linda Vixner[a], Renée Flacking[a], Marie Klingberg-Allvin[a], Ulla-Karin Schön[b] & Raziye Salari[c]

[a] *School of Health and Welfare, Dalarna University, Falun, Sweden*

[b] *Department of Social Work, Stockholm University, Stockholm, Sweden*

[c] *Child Health and Parenting (CHAP), Department of Public Health and Caring Sciences, Uppsala University, Uppsala, Sweden*

Corresponding author: Fatumo Osman, Dalarna University, 79188 Falun, Sweden, Telephone work: +4623778220, Cell phone: +46701918604, Email: fos@du.se

For peer review only

ABSTRACT

Objectives: This study aimed to evaluate the long-term impact (three-year follow-up) of a culturally tailored parenting support program (Ladnaan) on the mental health of Somali-born parents and their children living in Sweden.

Methods: In this longitudinal cohort study, Somali-born parents with children aged 11–16 were followed up three years after they had participated in the Ladnaan intervention. The Ladnaan intervention comprises two main components: societal information, and the Connect parenting program delivered using a culturally sensitive approach. It consists of 12 weekly group-based sessions each lasting 1–2 hours. The primary outcome was improved mental health in children, as measured by the Child Behavior Checklist (CBCL). The secondary outcome was improved mental health in parents, as measured by the General Health Questionnaire (GHQ-12). Data were collected from the parent's perspective.

Results: Of the 60 parents who were originally offered the intervention, 51 were included in this long-term follow-up. The one-way repeated measures (baseline to the three-year follow-up) Analysis of Variance (ANOVA) for the CBCL confirmed maintenance of all the treatment gains for children: total problem scores (95% *CI*, 11.49–18.00, $d = 1.57$), and externalizing problems (95% CI, 2.48–5.83, $d = 0.86$). Similar results were observed for the parents' mental health (95% CI, 0.40–3.11, $d = 0.46$).

Conclusion: Positive changes in the mental health of Somali-born parents and their children were maintained three years after they had participated in a parenting support program that was culturally tailored and specifically designed to address their needs. Our findings highlight the long-term potential benefits of these programs in tackling mental health issues in immigrant families.

For peer review only

Clinical Trial: The original randomized controlled trial has been registered at www.clinicaltrials.gov (identifier NCT02114593). In the current study, we focus on the intervention group only.

ARTICLE SUMMARY

Strengths and limitations of the study

- The research used a longitudinal cohort study design (three-year follow-up) of an intervention group who received support from a culturally tailored parenting program.
- The majority of the original sample were retained in this three-year follow-up.
- The main limitation of the study was the lack of a control group, which only allowed within-group analyses.
- The control group was offered the intervention shortly after the two-month follow-up due to ethical and practical reasons.
- Data were collected through parental self-report.

For peer review only

INTRODUCTION

Over the past 20 years, Sweden and many other European countries have experienced a rapid increase in immigration, particularly immigrants who have fled from areas of war. [1] Stress factors due to immigration have a negative influence on the mental health of parents and their children. [2-7] More specifically, studies have shown that post-migration factors such as lack of social networks and support, perceived discrimination in the host county, inadequate accommodation, and acculturation challenges contribute to behavioral problems in children. [7-13] Studies have also reported that the mental health problems of immigrant parents are associated with low confidence in parenting [14-16] and the use of authoritarian parenting, which has been shown to be related to children externalizing problems. [6, 17, 18]. In addition to negative parenting practices, power conflicts between parents and children are also associated with more behavioral problems among immigrant children. [17, 18].

There is a large body of evidence indicating that parenting support programs promote positive parenting practices and parent-child relationships, decrease children's internalizing and externalizing problems, and improve parents' mental health problems. [19, 20] However, parents with an immigrant background have reported a need for culturally tailored parenting support programs that assist them in adapting their parenting to the host country, improving their relationships with their children, [7, 21-24] and improving their children's mental health. [25] Few studies have attempted to culturally tailor parenting programs to immigrant families. These studies have reported that culturally tailored parenting programs improve children's mental health, strengthen parenting roles, and enhance parent-child relationships in the short-term. [26-29] However, little is known about the long-term effects of these programs on the mental health of parents and their children.

For peer review only

Objectives

This study aimed to evaluate the long-term impact (three-year follow-up) of a culturally tailored parenting support program (Ladnaan) on the mental health of Somali-born parents and their children living in Sweden.

METHODS

Study design

This cohort study is a longitudinal (three-year) follow-up of the intervention group in a randomized controlled trial [27, 28] which evaluated the impact of a culturally tailored parenting support program for Somali-born parents living in Sweden, referred to as the Ladnaan intervention. Ethical approval was obtained from the Swedish Regional Ethical Review Board in Uppsala, Sweden (Dnr 2014/048/2), and the trial was registered at www.clinicaltrials.gov (identifier NCT02114593).

Originally, the study included a two-month and a six-month post-intervention follow-up for both intervention and control groups. However, the participating municipality started offering the intervention to the control group shortly after the two-month follow-up data collection. As we had lost most of our control group by the sixth month, we opted for a three-year post-intervention follow-up instead.

Setting and participants

The study was conducted in a town in the middle of Sweden with approximately 50,000 inhabitants, of whom approximately 3,000 are of Somali origin. Baseline (pre-intervention) and two-month follow-up data were collected between May 2014 and October 2015. The three-year post-intervention data were collected between April and October 2018. Data were

For peer review only

collected by the first author and by research assistants who were trained in the recruitment process, taking informed consent, and the use of the instruments employed in the study.

The main trial included 120 Somali-born parents with children aged 11–16 years and self-perceived stress related to parenting practices. If the parents had more than one child aged 11–16, they were asked to complete the questionnaire for the child with whom they particularly wished to improve their relationship. In two-parent families, both parents were offered the possibility of participating in the intervention, but, throughout the study, only the parent who was screened at baseline completed the questionnaire. Originally, we had also planned to collect data from the children. However, the parents did not consent to their children's direct participation in the study (mainly due to the fear of Social Services). Thus, only parent-reported data were available.

Baseline data were collected prior to randomization and the follow-up data were collected two months as well as three years post-intervention. In comparison to the parents in the control group, the parents in the intervention group reported significant improvement in their children's and their own mental health at the two-month follow-up. [27, 28] Since most of the parents in the control group received the intervention soon after the two-month follow-up, the present paper focuses on the three-year follow-up of the original intervention group. Of the 60 parents in the intervention group who completed the baseline assessment, three were lost to the two-month follow-up, and six were lost to the three-year follow-up. Thus, 51 parents in the intervention group were followed up three years post-intervention (see Figure 1).

[Insert Figure 1 here]

Patient and Public involvement statement

A reference group consisting of key persons from the Somali community, Somali associations, Social Services, School Health and other stakeholder organisations were

engaged in our original study. The reference group has given feedback on the initial design, conducting the project and disseminating the results.

Intervention

The Ladnaan intervention, developed based on a qualitative study conducted prior to the trial, [7] consists of two main components: societal information and the Connect parenting program. [30] It included 12 weekly sessions with groups of 12–17 parents, each lasting approximately 1–2 hours.

The first two sessions covered societal information relevant to Somali-born parents, including an overall view of the Swedish Child Welfare Services, the Convention on the Rights of the Child, and parenting styles in Sweden. The societal information component was developed through a qualitative study conducted with Somali-born parents that aimed to explore Somali-born parents' need for parenting support. [7] The other 10 sessions covered the Connect parenting support program, which is a standardized program based on attachment theory. The Connect program addresses nine different principles of child development, parent–child relationships, and challenging interactions, and included role playing, case examples, and reflection exercises. [30] (Detailed information of each session is shown in online supplemental table S1). The cultural sensitivity delivery approach was employed to ensure the sustainability of the parenting program after the study was finalized. This delivery approach incorporated elements, such as delivering the program in the participants' native language, using group leaders of similar background and experience, and culturally tailoring the role playing and case examples to make them understandable and relatable for the participants. Only the parenting style theme in the societal information sessions was delivered by a Swedish-speaking professional from the Family and Child Welfare Services and interpreted by one of the group leaders.

For peer review only

In connection with every session, the parents were offered support for reading and writing letters from the authorities, such as the migration agency, social welfare services and hospitals.

Outcome measures

The CBCL for children aged 6 to 18 years (CBCL 6–18) [30] was used to measure the children's mental health from the parents' perspective. We opted to use the CBCL 6–18 even for the children who had already turned 18 at the three-year follow-up because: (1) all the children were still living at home with their parents and (2) using the adult version would have led to difficulties in estimating the changes in the level of problems from baseline. The CBCL 6–18 consists of 133 items encompassing two sections: (1) competence scales (20 items on activities and social scales) and (2) emotional and behavioral problems (113 items). In this study, only items related to emotional and behavioral problems were used. According to the Achenbach System of Empirically Based Assessment (ASEBA) guideline [31], alcohol- and sex-related items can be excluded for Muslim respondents. In this study, five sex-related items were considered culturally inappropriate for our target population and thus, they were omitted, leaving 107 items in the CBCL. There were three possible response options for each problem item: 0 (not true), 1 (somewhat or sometimes true), and 2 (very true or often true). Using the ASEBA manual for CBCL 6–18 [31], we constructed six variables: a total problem score, two broad groupings of emotional and behavioral problems (internalizing and externalizing problems), and three syndrome scales (social, thought, and attention problems). Higher scores indicate greater problem severity. In the current sample, Cronbach's alphas at baseline, two-month follow-up and three-year follow-up were respectively .82, .75, and .69 for total problem scores and .85, 73, and .75 for externalizing problems. Cronbach's alphas for internalizing problems and the three syndrome scales were low (for a detailed report

For peer review only

please see Supplementary Table 2S) and thus we opted to report the results for them only in Supplementary Table 3S and Figure 1S.

The 12-item version of the GHQ-12 [32] was used to measure the parents' overall mental health over the previous few weeks. Each item was rated on a four-point Likert scale from 1 (better than usual) to 4 (much worse than usual), with higher scores indicating higher mental health distress. Cronbach's alphas for GHQ-12 in this study were .80 (baseline), .91 (two-month follow-up) and .81 (three-year follow-up).

Both CBCL 6–18 and GHQ-12 were translated into Somali following the five steps of the World Health Organization's process of translation. [33] The translation started with forward translation (from English to Somali) by the first author and an interpreter who worked independently. After the translations were compared, a reconciled version was agreed upon. In the second step, backward translation was conducted by a bilingual social worker. In the third step, the backward translation was compared with the original version and then with the Somali version to check if the forward translation was accurate. In the fourth step, the Somali version of CBCL 6–18 and GHQ-12 were pilot tested with three mothers and fathers for accuracy. In the last step, the revision was proofread and approved by the ASEBA for CBCL 6–18 and by Mapi Research Trust for GHQ-12.

Statistical methods

All analyses were performed using IBM SPSS Statistics (Version 26). There were a few cases of missing data at the item level because some participants failed to answer all the questions. Thus, we started the analysis by reconstructing the mean outcome scores for participants who had at least filled in 70% of the items on each scale or subscale. This resulted in the retention of all 51 cases for CBCL and 49 cases for GHQ-12 in the analyses.

For peer review only

Descriptive statistics of baseline data were presented with mean (*SD*) or proportions (%). We used a series of t-tests and chi-square tests to compare the background characteristics of those who remained in the study at the three-year follow-up and those who dropped out. One-way repeated measure Analysis of Variances (ANOVAs) were used to compute the within-subject change over time from baseline to two-month follow-up and three-year follow-up. We started by performing omnibus tests (whether the three means were different overall). When omnibus tests were significant ($p < .05$), we carried out pairwise tests to specifically examine differences from baseline to two-month follow-up and from baseline to three-year follow-up. During these post-hoc analyses, we adjusted the *p* values for multiple comparison using Bonferroni method. Cohen's *d* effect size was calculated from *F* tests ($d = 0.2$, small; $d = 0.5$, medium; and $d = 0.8$, large). [34]

In addition, the clinical significance of change was measured from baseline to two-month follow-up and three-year follow-up using the method recommended by Jacobson and Truax. [35] Population norms for the CBCL 6–18 and GHQ-12 were not available for the present study population. Therefore, we used the pretest scores for all participants in the original randomized controlled trial to calculate the standard error of differences (S_{diff}). We assumed a measurement reliability of 0.8 for each measure. The clinical significance of change was then presented as the proportion of children/parents who had deteriorated, remained unchanged, or improved at two-month and three-year follow-ups.

For peer review only

RESULTS

Attrition and baseline characteristics

Table 1 presents baseline characteristics of the participants who remained in the study at the three-year follow-up and those who dropped out. No statistically significant differences were found between the two groups. Of the 51 participants who remained at the follow-up and were included in the analyses, 34 were mothers and 17 were fathers. Only five of them were employed at the time. Most of them had not entered upper secondary school, were married and living with their partner. The six participants who dropped out of the study at the three-year follow-up were all mothers, were educated up to upper secondary school level only, and were unemployed. Five of these six were not cohabiting with a partner.

A majority of the parents (n=40) attended at least eight of the 12 intervention sessions. Most parents also received support from the group leaders in the form of reading letters from government agencies. However, this information was not formally recorded and thus we do not have the exact number of parents who used this service.

[Insert Table 1 here]

Long-term findings

Although most variables were not normally distributed, ANOVA is considered relatively robust to violations of normality assumptions. [36, 37]

Mauchly's test indicated that the assumption of sphericity had been violated for all the three outcomes, therefore following the recommendations by Howell, [38] we report multivariate tests (Pillai's Trace). The results show significant improvement over time for all outcomes (see Table 2 for means, standard deviations, mean differences, *F* values, and 95% confidence intervals [*CI*s] and effect sizes).

For peer review only

For parent-reported CBCL, repeated measure ANOVA revealed significant improvements in children from baseline to the three-year follow-up for both total problem scores (95% *CI*, 11.49–18.00), and externalizing problems (95% *CI*, 2.48–5.83). The effect sizes were all large. Similarly, significant improvements were observed for parents' mental health as measured by GHQ-12. The associated effect sizes were medium.

[Insert Table 2 here]

Clinical significance of change in children's and parents' mental health

Figure 2 shows the clinical significance of change in the mental health of children and parents at two-month and three-year follow-ups. The proportion of children who exhibited clinically significant change from baseline to the two-month follow-up was 80% for total problem score and 82% for externalizing problems. This indicated that the level of problems remained unchanged for most children. Very few children exhibited negative changes (4% and 6%), while positive changes were observed for a higher number of children (16% and 12%).

The positive changes were more pronounced from baseline to the three-year follow-up: 51% of children demonstrated clinically significant improvement on total problem score and 26% on externalizing problems. No negative changes were observed for children in either of the outcomes.

Most parents showed no clinically significant changes from baseline to the two-month or three-year follow-ups (75% and 86%, respectively). Very few showed negative changes (6% and 2% at the two-month and three-year follow-ups, respectively), while positive changes were observed for a higher number of parents (20% and 12% at the two-month and three-year follow-ups, respectively).

[Insert Figure 2 here]

For peer review only

Supplementary and sensitivity analyses. Long-term follow-up data were available for 36 out of 39 parents in the control group who had received the intervention. One-way repeated measure ANOVAs on this group revealed similar significant improvement from baseline to the long-term follow-up for all the outcomes. In addition, we imputed the data for the six participants who had dropped out from the study using the worst available scores (i.e., worst case scenario). One-way repeated measure ANOVAs on the imputed data revealed similar significant improvement from baseline to the long-term follow-up for all the outcomes. These results point to the robustness of the main findings.

For peer review only

DISCUSSION

To the best of our knowledge, this is the first study to evaluate the long-term effect of a culturally tailored parenting support program on the mental health of immigrant parents and their children. Our findings reveal that all the short-term positive changes in the mental health of children and parents were maintained three years post-intervention. The findings also suggest that clinically positive changes were generally retained over time.

Our findings are in line with previous systematic and meta-analytical reviews, [39, 40] which have indicated that positive change in children's emotional and behavioral problems is sustained over time. However, the parenting programs evaluated in these previous studies were mainly based on cognitive behavioral theories. In contrast, we tested the Connect program in our study, which is based on attachment theory. Our findings are also consistent with another Swedish study on non-immigrant parents that showed that the Connect program had continuously improved children's behavioral and emotional problems two years after the intervention [41], although the related effect sizes were generally larger in our study.

The improvement in children's mental health can be explained by parents' reflectivity and sensitivity towards their children's emotional needs, which is the aim of the Connect program. [42] Our previous qualitative study [43] revealed that after participating in the Connect program, parents became more aware of their children's expressed and non-expressed needs and made themselves more emotionally available to them. Parents also reported using the principles in the Connect program, such as 'taking a step back'. [43] The positive results can also be attributed to factors unrelated to the intervention. For example, as children grow older, teenage problems and parent-adolescent conflicts diminish, meaning parents report fewer problems.

For peer review only

In this study, a smaller change was detected in parents' mental health. Although parenting support programs do not aim to improve parents' mental health directly, studies have shown that parents can benefit from parenting support programs that are tailored to their needs. [44-46] However, other external factors (such as employment, acculturation, perceived discrimination, and social isolation) might play a more important role in parental mental health. [7, 12, 13, 47, 48] Therefore, the benefits associated with participation in parenting programs might be more difficult to maintain over time.

Strengths and limitations

Several strengths and limitations of this study should be taken into consideration. First, there are few studies on the long-term effect of parenting programs, particularly those delivered to immigrant parents. [49] Second, most studies with long-term follow-ups have high-dropout rates. In our study, we were successful in retaining 85% of our original sample.

The main limitation of the study was the lack of a control group, which only allowed within-group analyses. The control group was offered the intervention shortly after the two-month follow-up due to ethical and practical reason. The collaborator municipality was resolved that the intervention should be given to the parents in the control group because of its short-term positive impact on participating families.

Another limitation is that the outcome measures used in this study were not previously tested for their reliability and validity on this specific population. In the current sample, internal consistency was low for some of the CBCL subscales. However, both CBCL and GHQ are well-established questionnaires that have been readily used in cross-cultural settings showing good reliability and validity [32, 50]. Thus, it is important to examine Somali parents understanding of the individual items and also test the reliability and validity of these two measures, particularly CBCL, in a larger sample of Somali parents.

For peer review only

Finally, similar to many other studies, the intervention was evaluated using parental reports only, which may be biased. It is preferable to collect data from parents, children, and teachers. In our study, parents might have had reservations about reporting problems, as highlighted in previous studies. [7, 22] We found that parents reported a very low number of problems at the three-year follow-up. However, this can be normative for children at this age, or it might indicate that attending the program changed parental perceptions of their children's behavior. Including children's self-reports would have strengthened the results. We had, in fact, initially intended to evaluate the intervention using both parents' and children's reports. However, the vast majority of the participating parents refused to consent to their children's direct participation in the study. This was mainly due to their (mis)perception of child protection policies in Sweden; they were afraid that the children might, accidently or intentionally, say something true or untrue that would lead to the involvement of Family and Child Welfare Services and their children would potentially be taken away. Interestingly, at the three-years follow-up, parents readily agreed with their children participation in a focus group discussion. This implies that addressing mis-conceptions about child protection policies and building a long-term relationship with immigrant communities might facilitate the issues regarding children's participation in research studies.

Conclusion and implication for clinical practice

Previous studies have shown that participation in a culturally tailored parenting program was associated with positive changes in the mental health of Somali-born parents and their children. In the current study, we showed that these positive changes were maintained three years post-intervention. Given the study's limitations, our findings need to be replicated. Future studies should strive to measure the long-term effects of parenting programs for immigrant parents using a control group, and by including well-validated ratings from parents, children and teachers. Despite the reported limitations, the study highlights the

For peer review only

potential benefits of offering a culturally tailored parenting support program to immigrant families.

Acknowledgement

The authors would like to thank all the parents who participated in this study. We also thank research assistant Abdikerim Mohamed for his important work in the data collection, and the Kamprad Foundation for funding this study.

Competing Interests

The authors have no conflicts of interest relevant to this article to disclose.

Funding and all other required statements

This work was supported by the Kamprad Foundation, grant number 2017:0069.

Contributors' statement

All authors conceptualized and designed the study.

Dr Osman, Dr Vixner, and Dr Salari were responsible for data analyses and interpretation. They drafted the initial manuscript, reviewed, revised, and approved the final manuscript as submitted.

Dr Flacking reviewed the data analyses and interpretation. She revised, reviewed, and approved the final manuscript as submitted.

Dr Schön reviewed, revised, and approved the final manuscript as submitted.

Dr Klingberg-Allvin reviewed, revised, and approved the final manuscript as submitted.

All authors approved the final manuscript as submitted.

For peer review only

Ethics approval:

The Swedish Regional Ethical Review Board in Uppsala, Sweden (Dnr 2014/048/2).

Data sharing statement

The data sets for this study is not open access, but can be made available upon request to the Principal Investigator (FO) and according to the ethical approval.

For peer review only

REFERENCES

1. Eurostat. Migration and migrant population statistics. https://ec.europa.eu/eurostat/statistics-explained/index.php/Migration_and_migrant_population_statistics#Migrant_population:_22.3_million_non-EU_citizens_living_in_the_EU_on_1_January_2018. Published 2019. Accessed 20 October, 2019.
2. Bhugra D. Migration and mental health. *Acta Psychiatri Scand.* 2004;109(4):243-258.
3. Gilliver SC, Sundquist J, Li X and Sundquist K. Recent research on the mental health of immigrants to Sweden: a literature review. *Eur J Public Health.* 2014;24 Suppl 1:72-79.
4. Kirmayer LJ, Narasiah L, Munoz M, et al. Common mental health problems in immigrants and refugees: general approach in primary care. *Can. Med. Assoc. J.* 2011;183(12):E959-E967.
5. Kouider EB, Koglin U and Petermann F. Emotional and Behavioral Problems in Migrant Children and Adolescents in American Countries: A Systematic Review. *J. Immigr. Minor Health.* 2015;17(4):1240-1258.
6. Nguyen H, Rawana JS and Flora DB. Risk and Protective Predictors of Trajectories of Depressive Symptoms Among Adolescents from Immigrant Backgrounds. *J. Youth Adolesc.* 2011;40(11):1544-1558.
7. Osman F, Klingberg-Allvin M, Flacking R and Schön U-K. Parenthood in transition – Somali-born parents' experiences of and needs for parenting support programmes. *BMC Int. Health Hum. Rights.* 2016;16(7):1-11.
8. Liebkind K and Jasinskaja-Lahti I. Acculturation and Psychological Well-Being among Immigrant Adolescents in Finland: A Comparative Study of Adolescents From Different Cultural Backgrounds. *J. Adolesc. Res.* 2000;15(4):446-469.
9. Lincoln AK, Lazarevic V, White MT and Ellis BH. The Impact of Acculturation Style and Acculturative Hassles on the Mental Health of Somali Adolescent Refugees. *J Immigr Minor Health.* 2015.
10. Lindencrona F, Ekblad S and Hauff E. Mental health of recently resettled refugees from the Middle East in Sweden: the impact of pre-resettlement trauma, resettlement stress and capacity to handle stress. *Soc. Psychiatry Psychiatr. Epidemiol.* 2008;43(2):121-131.
11. Miller KE and Rasmussen A. War exposure, daily stressors, and mental health in conflict and post-conflict settings: Bridging the divide between trauma-focused and psychosocial frameworks. *Social Science & Medicine.* 2010;70(1):7-16.
12. Warfa N, Bhui K, Craig T, et al. Post-migration geographical mobility, mental health and health service utilisation among Somali refugees in the UK: a qualitative study. *Health Place.* 2006;12(4):503-515.
13. Warfa N, Curtis S, Watters C, Carswell K, Ingleby D, Bhui K. Migration experiences, employment status and psychological distress among Somali immigrants: a mixed-method international study. *BMC Public Health.* 2012;12(1):1-12.
14. Dix T and Meunier LN. Depressive symptoms and parenting competence: An analysis of 13 regulatory processes. *Dev Rev.* 2009;29(1):45-68.
15. Gelkopf M and Jabotaro SE. Parenting style, competence, social network and attachment in mothers with mental illness. *Child Fam Soc Work.* 2013;18(4):496-503.
16. Haslam DM, Pakenham KI and Smith A. Social support and postpartum depressive symptomatology: The mediating role of maternal self-efficacy. *Infant Ment Health J.* 2006;27(3):276-291.

For peer review only

17. Park IJK, Kim PY, Cheung RYM and Kim M. The Role of Culture, Family Processes, and Anger Regulation in Korean American Adolescents' Adjustment Problems. *Am. J. Orthopsychiat.* 2010;80(2):258-266.
18. Stevens G, Vollebergh WAM, Pels TVM and Crijnen AAM. Parenting and internalizing and externalizing problems in Moroccan immigrant youth in the Netherlands. *J. Youth Adolesc.* 2007;36(5):685-695.
19. Stewart-Brown, S., et al., Impact of a general practice based group parenting programme: quantitative and qualitative results from a controlled trial at 12 months. *Arch Dis Child* 2004;89(6):519-525.
20. Morgan, Z., et al., The effects of parent-child relationships on later life mental health status in two national birth cohorts. *Soc Psychiatry Psychiatr Epidemiol* 2012;47(11):1707-1715.
21. Bowie BH, Wojnar D and Isaak A. Somali Families' Experiences of Parenting in the United States. *West. J. Nur. Res.* 2017;39(2):273-289.
22. Lewig K, Arney F and Salveron M. Challenges to parenting in a new culture: Implications for child and family welfare. *Eval. Program Plann.* 2010;33(3):324-332.
23. Renzaho AMN, Green J, Mellor D and Swinburn B. Parenting, family functioning and lifestyle in a new culture: the case of African migrants in Melbourne, Victoria, Australia. *Child Fam Soc Work.* 2011;16(2):228-240.
24. Renzaho AMN, McCabe M and Sainsbury WJ. Parenting, role reversals and the preservation of cultural values among Arabic speaking migrant families in Melbourne, Australia. *Int. J. Intercult. Relat.* 2011;35(4):416-424.
25. Aronsson AP, Warner G and Sarkadi A, Osman F. "I'm a mother who always tries to give my children hope"- Refugee women's experiences of their children's mental health. *Front. Psychiatry.* 2019;10:789.
26. Bjorknes R and Manger T. Can Parent Training Alter Parent Practice and Reduce Conduct Problems in Ethnic Minority Children? A Randomized Controlled Trial. *Prev. Sci.* 2013;14(1):52-63.
27. Osman F, Flacking R, Schön U-K and Klingberg-Allvin M. A Support Program for Somali-born Parents on Children's Behavioral Problems. *Pediatrics.* 2017;139(3).
28. Osman F, Salari R, Klingberg-Allvin M, Schön U-K and Flacking R. Effects of a culturally tailored parenting support programme in Somali-born parents' mental health and sense of competence in parenting: a randomised controlled trial. *BMJ Open.* 2017;7(12).
29. Renzaho AM and Vignjevic S. The impact of a parenting intervention in Australia among migrants and refugees from Liberia, Sierra Leone, Congo, and Burundi: Results from the African Migrant Parenting Program. *J. Fam. Stud.* 2011;17(1):71-79.
30. Moretti MM, Braber K and Obsuth I. *Connect. An Attachment-focused treatment group for parents and caregivers–A principle based manual.* Burnaby, British Columbia, Canada: Simon Fraser University.; 2009.
31. Achenbach TM and Rescorla LA. *Manual for the ASEBA School-Age Forms & Profiles.* Burlington, VT: University of Vermont, Research Center for Children, Youth, & Families; 2001.
32. Goldberg DP, Gater R, Sartorius N, et al. The validity of two versions of the GHQ in the WHO study of mental illness in general health care. *Psychol. Med.* 1997;27:191-197.
33. World Health Organization W. Process of translation and adaptation of instruments. In: World Health Organazation; 2013.
34. Cohen J. A power primer. *Psychol. Bull.* 1992;112(1):155-159.

For peer review only

35. Jacobson NS and Truax P. Clinical significance: a statistical approach to defining meaningful change in psychotherapy research. *J. Consult. Clin. Psychol.* 1991;59(1):12.
36. Harwell M.R., et al., Summarizing Monte Carlo Results in Methodological Research: The One- and Two-Factor Fixed Effects ANOVA Cases. J. Educ. Stat. 1992. 17(4): 315-339.
37. Lix, L.M., J.C. Keselman, and H.J. Keselman, Consequences of Assumption Violations Revisited: A Quantitative Review of Alternatives to the One-Way Analysis of Variance "F" Test. Rev. Educ. Res. 1996. 66(4): 579-619.
38. Howell, D.C. (2009). *Statistical Methods for Psychology* (5th ed.). Pacific Grove CA: Duxbury.
39. Lundahl B, Risser H and Lovejoy M. A meta-analysis of parent training: moderators and follow-up effects. *Clin Psychol Rev.* 2006;26:86 - 104.
40. van Aar J, Leijten P, Orobio de Castro B and Overbeek G. Sustained, fade-out or sleeper effects? A systematic review and meta-analysis of parenting interventions for disruptive child behavior. *Clin. Psychol. Rev.* 2017;51:153-163.
41. Högström J, Olofsson V, Özdemir M, Enebrink P and Stattin H. Two-year findings from a national effectiveness trial: effectiveness of behavioral and non-behavioral parenting programs. *J. Abnorm. Child Psychol.* 2017;45(3):527-542.
42. Moretti MM and Obsuth I. Effectiveness of an attachment-focused manualized intervention for parents of teens at risk for aggressive behaviour: The Connect Program. *J. Adolesc.* 2009;32(6):1347-1357.
43. Osman F, Flacking R, Klingberg Allvin M and Schön U-K. Qualitative study showed that a culturally tailored parenting programme improved the confidence and skills of Somali immigrants. *Acta Paediatr.* 2019;108(8):1482-1490.
44. Griner D and Smith TB. Culturally adapted mental health interventions: A meta-analytic review. *Psychotherapy.* 2006;43(4):531-548.
45. Koerting J, Smith E, Knowles MM, et al. Barriers to, and facilitators of, parenting programmes for childhood behaviour problems: a qualitative synthesis of studies of parents' and professionals' perceptions. *Eur Child Adolesc Psych.* 2013;22(11):653-670.
46. Wells MB, Sarkadi A and Salari R. Mothers' and fathers' attendance in a community-based universally offered parenting program in Sweden. *Scand J Public Health.* 2015.
47. Grant J and Guerin PB. Applying Ecological Modeling to Parenting for Australian Refugee Families. *J. Transcult. Nurs.* 2014;25(4):325-333.
48. Missinne S and Bracke P. Depressive symptoms among immigrants and ethnic minorities: a population based study in 23 European countries. *Soc. Psychiatry Psychiat. Epidemiol.* 2012;47(1):97-109.
49. Özdemir M. How much do we know about the long-term effectiveness of parenting programmes? Advances, shortcomings, and future directions. *J. Child. Serv.* 2015;10(2):120-132.
50. Dumenci, L., et al., *Measurement Structure of the Turkish Translation of the Child Behavior Checklist Using Confirmatory Factor Analytic Approaches to Validation of Syndromal Constructs.* J. Abnorm Child Psychol, 2004. 32(3): p. 335-340.

For peer review only

Table 1. Characteristics of intervention participants at baseline for those who remained in the study (n = 51) and those who dropped out after two-month follow-up (n = 6)

	Remained		Dropped out	
**Categorical variables**	***n***	**%**	***n***	**%**
Parents				
Mothers	34	66.7	6	100.0
Fathers	17	33.3	0	0.0
Identified child's gender				
Girls	32	62.7	2	33.3
Boys	19	37.3	4	66.7
Years in Sweden [a]				
1-5 years	20	39.2	1	16.7
6 years or more	31	60.8	5	83.3
Highest educational level				
Less than upper secondary school	29	56.9	6	100.0
Upper secondary school or more	22	43.1	0	0.0
Occupation				
Employed	5	9.8	0	0.0
Other	46	90.2	6	100.0
Civic status				
Married	33	64.7	4	66.7
Single	18	35.3	2	33.3
Cohabiting with partner				
Yes	30	58.8	1	16.7
No	21	41.2	5	83.3
Worries about own financial situation [b]				
Yes	17	34.0	3	50.0
No	33	66.0	3	50.0
**Continuous variables**	***M***	***SD***	***M***	***SD***
Identified child's age at baseline	13.51	1.61	14.17	2.14
Participants' age	43.80	7.77	46.33	11.08
Number of children living at home [c]	5.74	2.77	4.33	2.94

Note. No statistically significant differences were found between the two groups

For peer review only

[a] None of the parents in our sample had lived in any other Western country before arriving in Sweden. Typically, the journey from Somali to Sweden takes less than a year with some Somalis having to wait in Somali's neighboring countries before continuing their journey to Europe and resettling in Sweden; [b] n = 50 for those who remained in the study; [c] n = 49 for those who remained in the study

For peer review only

Table 2. Within-group comparison of intervention participants at baseline, two-month follow-up, and three-year follow-up (N = 51)

					Within-subject change							
					Baseline to 2-month FU				Baseline to 3-year FU			
	Baseline Mean (*SD*)	2-month FU Mean (*SD*)	3-year FU Mean (*SD*)	*F* (2, 49)[a]	Mean diff	*F* (1, 50)[b]	95% *CI*	*d*	Mean diff	*F* (1, 50)[b]	95% *CI*	*d*
**CBCL 6-18**												
Total problem score	15.71 (9.8)	9.49 (7.4)	0.96 (2.1)	95.86***	6.22	15.13***	2.26-10.18	0.54	14.75	125.84***	11.49-18.00	1.57
Externalizing problems	4.67 (5.2)	2.39 (3.5)	0.51 (1.4)	26.94***	2.28	8.28*	0.32-4.23	0.40	4.12	37.77***	2.48-5.83	0.86
**GHQ-12**[a]	20.00 (4.2)	17.80 (4.8)	18.22 (2.3)	5.46**	2.18	7.37*	0.19-4.18	0.41	1.76	10.28**	0.40-3.11	0.46

[a] *F*(2, 47) for GHQ-12

[b] *p* values adjusted for multiple comparison (Bonferroni); *F*(1, 48) for GHQ-12

CBCL 6-18 = Child Behaviour Checklist for children 6-18 years, GHQ = General Health Questionnaire; FU = follow-up

*** $p < .001$; ** $p < .01$; * $p < .05$

For peer review only

Figure 1: Participant flowchart throughout the study (grey marked area concerns the current study)

Figure 2. Clinical significance of change from baseline: proportion of children (CBCL) and parents (GHQ) showing reliable change (N= 51 for children and 49 parents)

For peer review only

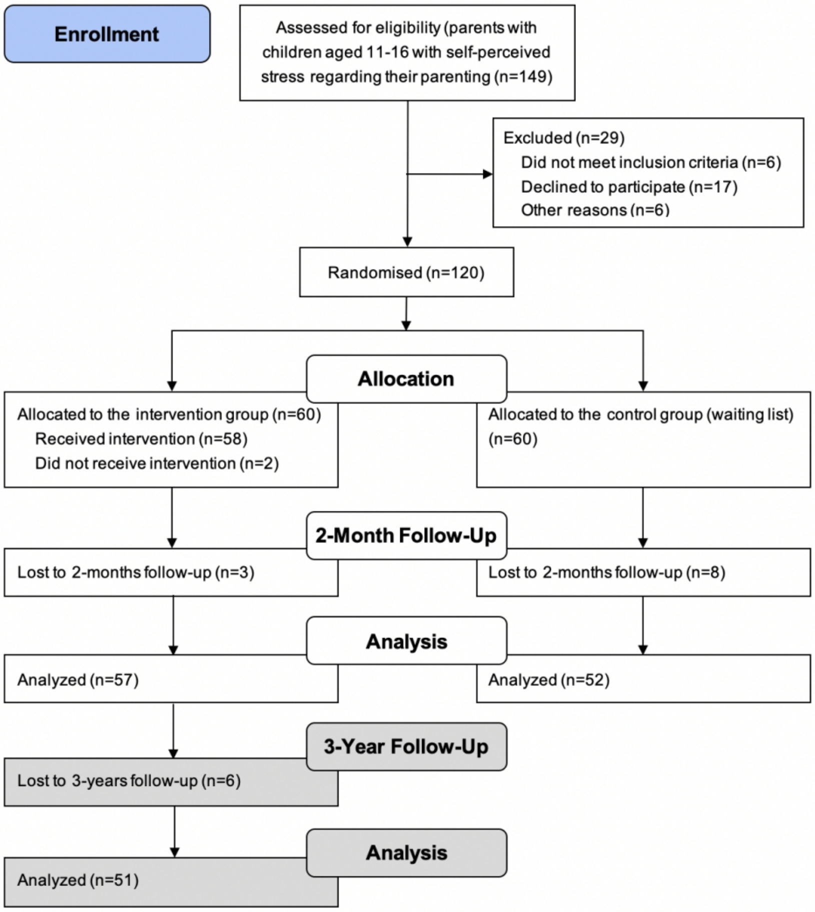

Figure 1. Participant flowchart throughout the study (grey marked area concerns the current study)

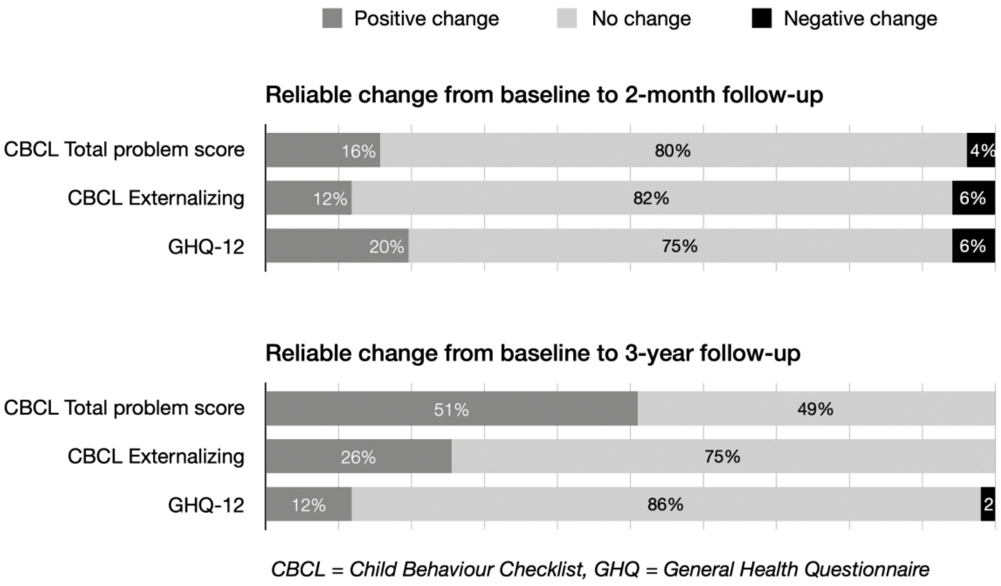

Figure 2. Clinical significance of change from baseline: proportion of children (CBCL) and parents (GHQ) showing reliable change (N =51 for children and 49 for parents)

483x364mm (144 x 144 DPI)

Supplementary

Supplemental Material for

The impact of a culturally tailored parenting program on the mental health of Somali parents and children living in Sweden: A longitudinal cohort study

Fatumo Osman[a,c], Linda Vixner[a], Renée Flacking[a], Marie Klingberg-Allvin[a], Ulla-Karin Schön[b] & Raziye Salari[c]

[a] *School of Education, Health and Social Studies, Dalarna University, Falun, Sweden*

[b] *Department of Social Work, Stockholm University, Stockholm, Sweden*

[c] *Child Health and Parenting (CHAP), Department of Public Health and Caring Sciences, Uppsala University, Uppsala, Sweden*

For peer review only

Supplementary

Table S1.

Content of the intervention: Connect program and societal information

Session	Intervention topics	Content of the session
	**Societal information**	
1	Child Welfare Services	- Knowledge and understanding on social service's work with children and youngsters.<br>- Various laws in the social services regarding the removal of the child from his/her family<br>- The support that social services have to offer for families
1	Parenting styles; authoritarian and democratic parenting	- Different parenting styles and the impact it has on child development<br>- Knowledge and understanding of parental responsibility regarding personal, economic and social growth<br>- Interaction between children and parents in the new context
2	Convention on the rights of the child	- Knowledge on the United Nations Convention on the Rights of the Child<br>- Reflection on how parents follow the child's right<br>- Reflection on having a child perspective
	- Connect program	
3	Introduction on Connect parenting program	- Introduction to Connect and the theory that the program is based on
4	All behaviour has a meaning	Children's behaviour and what it stands for, and how parents could recognize it
5	Attachment is for life	Skills to recognize the attachment needs of the infant, small children and teens
6	Conflict is a part of attachment	Conflict as a natural part of the relationship between parents and teens
7	Autonomous includes connection	The teens need regarding the autonomous while they want to be connected with their parents.
8	Empathy – the heartbeat of attachment	Practice and acknowledge empathy which is about being there for the child without condemning or giving solution of the problem.

For peer review only - http://bmjopen.bmj.com/site/about/guidelines.xhtml

Supplementary

9	Balance our needs with needs of others	Parents to recognize their own attachment needs and look for other sources of support than their children.
10	Growth and change are part of relationship	Parents to become aware of their past, and acknowledge what can promote and hinder the teens change and growth.
11	Celebrating attachment	Acknowledgement of that in every relationship there are both joy and pain which should be celebrated.
12	Two steps forward, One step back: staying on course	Understand on how to respond when the relationship is on turbulence and how doing that can establish a reconciliation and strengthen the relationship.

For peer review only

Supplementary

Table S2.

Internal consistency (Cronbach's alpha) for outcome measures at baseline, two-month follow-up, and three-year follow-up (N = 40-51)

	Number of items	Cronbach's alpha		
		Baseline	2-month FU	3-year FU
**CBCL 6-18**				
Total problem score	99	.82	.75	.69
Internalizing problems	32	.29	.62	.43
Externalizing problems	33	.85	.73	.75
Social problems	11	.29	.30	0
Thought problems	13	-.03	.21	.36
Attention problems	10	.75	.54	-.02
**GHQ-12**[a]	12	.80	.91	.81

CBCL 6-18 = Child Behaviour Checklist for children 6-18 years, GHQ = General Health Questionnaire; FU = follow-up

As shown in Table S2, the Cronbach's alpha for internalizing problems and the three syndrome scales were low and thus no further analyses were reported on these outcomes in the main paper. Below, we report the analyses for all the outcomes regardless of the level of Cronbach's alpha.

RESULTS

Long-term findings

Although most variables were not normally distributed, ANOVA is considered relatively robust to violations of normality assumptions [36, 37]

Mauchly's test indicated that the assumption of sphericity had been violated for five outcomes (CBCL total problem scores, externalizing, social problems and attention problems as well as GHQ-12), therefore following the recommendations by Howell, [38] we report multivariate tests (Pillai's Trace) for these five outcomes. The results show significant improvement over time for all outcomes (see Table S3 (compatible to Table 2 in the main paper) for means, standard deviations, mean differences, *F* values, and 95% confidence intervals [*CI*s] and effect sizes).

For parent-reported CBCL, repeated measure ANOVA revealed significant improvements in children for all primary outcomes from baseline to the three-year follow-up: total problem scores (95% *CI*, 11.49–18.00), internalizing problems (95% *CI*, 4.43–6.24), and externalizing problems (95% *CI*, 2.48–5.83). The effect sizes were all large. Similarly, significant improvements were observed for all secondary outcomes, i.e., CBCL scores for social, thought, and attention problems in children, and parents' mental health as measured by GHQ-12. The associated effect sizes for the secondary outcomes were medium to large.

For peer review only

Supplementary

Table S3.

Within-group comparison of intervention participants at baseline, two-month follow-up, and three-year follow-up (N = 51)

					Within-subject change							
					Baseline to 2-month FU				Baseline to 3-year FU			
	Baseline Mean (*SD*)	2-month FU Mean (*SD*)	3-year FU Mean (*SD*)	*F* (2, 49) [a]	Mean diff	*F* (1, 50) [b]	95% *CI*	*d*	Mean diff	*F* (1, 50) [b]	95% *CI*	*d*
**CBCL 6-18**												
Total problem score	15.71 (9.8)	9.49 (7.4)	0.96 (2.1)	95.86***	6.22	15.13***	2.26-10.18	0.54	14.75	125.84***	11.49-18.00	1.57
Internalizing problems	5.65 (2.6)	3.76 (2.9)	0.31 (0.8)	84.08***	1.88	15.98***	0.72-3.05	0.56	5.33	212.50***	4.43-6.24	2.04
Externalizing problems	4.67 (5.2)	2.39 (3.5)	0.51 (1.4)	26.94***	2.28	8.28*	0.32-4.23	0.40	4.12	37.77***	2.48-5.83	0.86
Social problems	1.65 (1.5)	0.76 (1.2)	0.02 (0.1)	35.95***	0.88	12.78**	0.27-1.49	0.50	1.63	61.44***	1.11-2.14	1.10
Thought problems	0.61 (0.8)	0.53 (0.9)	0.08 (0.3)	9.83***	0.08	0.29	-0.28-0.44	0.08	0.53	23.27***	0.26-0.80	0.68
Attention problems	1.23 (2.2)	0.61 (1.3)	0.04 (0.2)	11.80***	0.63	3.72	-0.18-1.43	0.27	1.20	15.59***	0.45-1.95	0.55
**GHQ-12**[a]	20.00 (4.2)	17.80 (4.8)	18.22 (2.3)	5.46**	2.18	7.37*	0.19-4.18	0.41	1.76	10.28**	0.40-3.11	0.46

[a] *F*(2, 47) for GHQ-12

[b] *p* values adjusted for multiple comparison (Bonferroni); *F*(1, 48) for GHQ-12

CBCL 6-18 = Child Behavior Checklist for children 6-18 years, GHQ = General Health Questionnaire; FU = follow-up

Grey marked indicate outcomes with low Cronbach's alpha

*** $p < .001$; ** $p < .01$; * $p < .05$

For peer review only

Supplementary

Clinical significance of change in children's and parents' mental health

Figure S1 shows the clinical significance of change in the mental health of children and parents at two-month and three-year follow-ups. The proportion of children who exhibited no clinically significant change from baseline to the two-month follow-up ranged from 61% on social problems to 88% on attention problems. This indicated that the level of various problems remained unchanged for most children. Very few children exhibited negative changes (4% to 8%), while positive changes were observed for a higher number of children (8% to 31%).

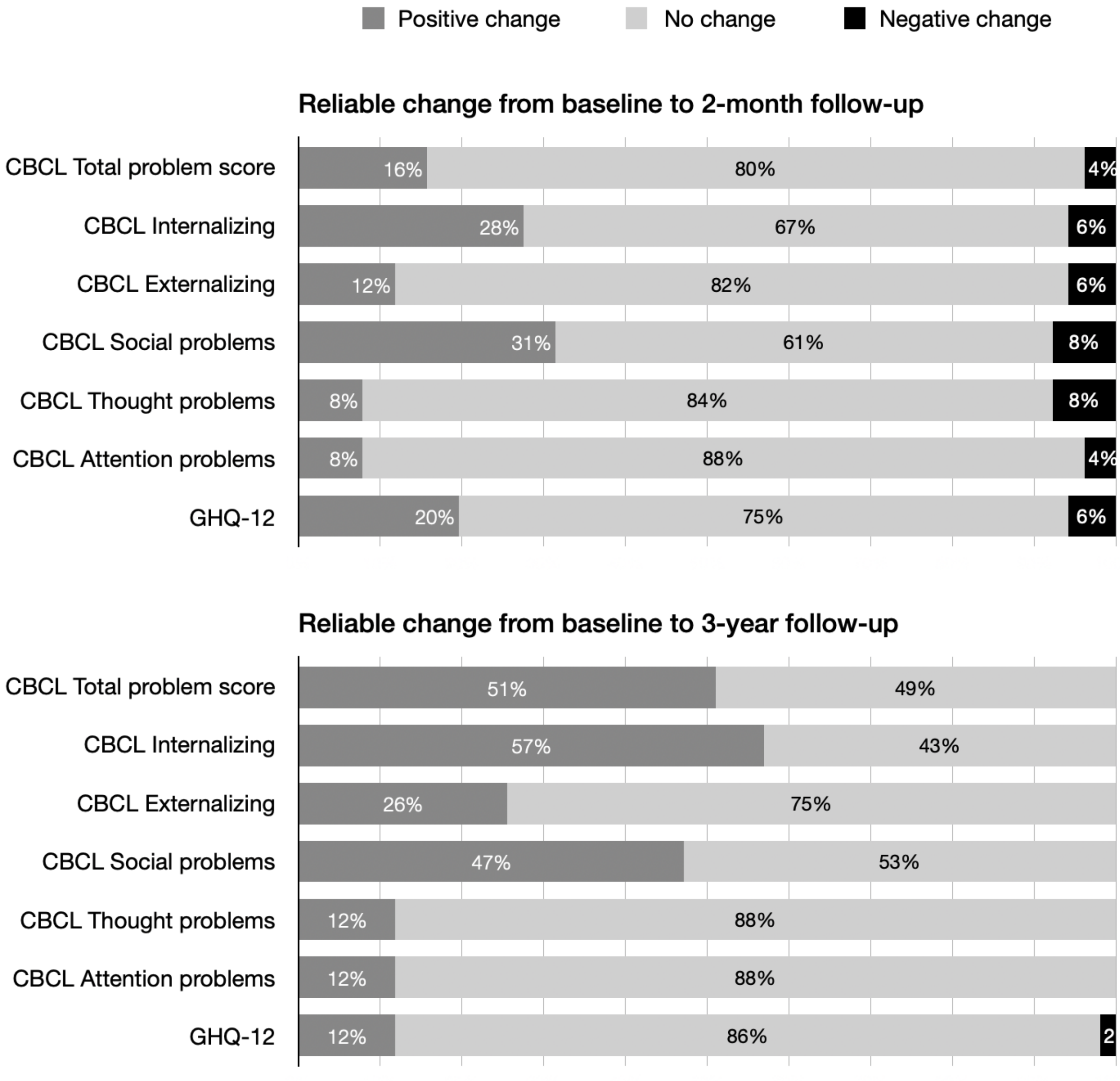

CBCL = Child Behaviour Checklist, GHQ = General Health Questionnaire

Figure S1. Clinical significance of change from baseline: proportion of children (CBCL) and parents (GHQ) showing reliable change (N =51 for children and 49 for parents)

Supplementary

The positive changes were more pronounced from baseline to the three-year follow-up: 25% to 57% of children demonstrated clinically significant improvement on the primary outcomes, while 12% to 47% demonstrated clinically significant improvement on the secondary outcomes. No negative changes were observed for children in any of the outcomes.

Most parents showed no clinically significant changes from baseline to the two-month or three-year follow-ups (71% and 82%, respectively). Very few showed negative changes (6% and 2% at the two-month and three-year follow-ups, respectively), while positive changes were observed for a higher number of parents (20% and 12% at the two-month and three-year follow-ups, respectively).

Supplementary and sensitivity analyses. Long-term follow-up data were available for 36 out of 39 parents in the control group who had received the intervention. One-way repeated measure ANOVAs on this group revealed similar significant improvement from baseline to the long-term follow-up for all the outcomes except CBCL attention problems, which had remained the same. In addition, we imputed the data for the six participants who had dropped out from the study using the worst available scores (i.e., worst case scenario). One-way repeated measure ANOVAs on the imputed data revealed similar significant improvement from baseline to the long-term follow-up for all the outcomes. These results point to the robustness of the main findings.

REFERENCES

36. Harwell M.R., et al., Summarizing Monte Carlo results in methodological research: The one- and two-factor fixed effects ANOVA cases*. J. Educ. Stat.* 1992. 17(4): 315-339.
37. Lix, L.M., J.C. Keselman, and H.J. Keselman, Consequences of assumption violations revisited: A quantitative review of alternatives to the one-way analysis of variance "F" test. *Rev. Educ. Res.* 1996. 66(4): 579-619.
38. Howell, D.C. (2009). *Statistical Methods for Psychology* (5th ed.). Pacific Grove CA: Duxbury.

For peer review only

STROBE 2007 (v4) Statement—Checklist of items that should be included in reports of *cohort studies*

Section/Topic	Item #	Recommendation	Reported on page #
**Title and abstract**	1	(*a*) Indicate the study's design with a commonly used term in the title or the abstract	1
		(*b*) Provide in the abstract an informative and balanced summary of what was done and what was found	2
**Introduction**			
Background/rationale	2	Explain the scientific background and rationale for the investigation being reported	4
Objectives	3	State specific objectives, including any prespecified hypotheses	5
**Methods**			
Study design	4	Present key elements of study design early in the paper	5
Setting	5	Describe the setting, locations, and relevant dates, including periods of recruitment, exposure, follow-up, and data collection	5-6
Participants	6	(*a*) Give the eligibility criteria, and the sources and methods of selection of participants. Describe methods of follow-up	5-6
		(*b*) For matched studies, give matching criteria and number of exposed and unexposed	n/a
Variables	7	Clearly define all outcomes, exposures, predictors, potential confounders, and effect modifiers. Give diagnostic criteria, if applicable	7-9
Data sources/ measurement	8*	For each variable of interest, give sources of data and details of methods of assessment (measurement). Describe comparability of assessment methods if there is more than one group	7-9
Bias	9	Describe any efforts to address potential sources of bias	9
Study size	10	Explain how the study size was arrived at	6
Quantitative variables	11	Explain how quantitative variables were handled in the analyses. If applicable, describe which groupings were chosen and why	9-10
Statistical methods	12	(*a*) Describe all statistical methods, including those used to control for confounding	9-10
		(*b*) Describe any methods used to examine subgroups and interactions	9-10
		(*c*) Explain how missing data were addressed	9
		(*d*) If applicable, explain how loss to follow-up was addressed	11-12
		(*e*) Describe any sensitivity analyses	13
**Results**			

For peer review only - http://bmjopen.bmj.com/site/about/guidelines.xhtml

Participants	13*	(a) Report numbers of individuals at each stage of study—eg numbers potentially eligible, examined for eligibility, confirmed eligible, included in the study, completing follow-up, and analysed	11
		(b) Give reasons for non-participation at each stage	11
		(c) Consider use of a flow diagram	Figure 1
Descriptive data	14*	(a) Give characteristics of study participants (eg demographic, clinical, social) and information on exposures and potential confounders	11
		(b) Indicate number of participants with missing data for each variable of interest	11
		(c) Summarise follow-up time (eg, average and total amount)	11
Outcome data	15*	Report numbers of outcome events or summary measures over time	11-12
Main results	16	(*a*) Give unadjusted estimates and, if applicable, confounder-adjusted estimates and their precision (eg, 95% confidence interval). Make clear which confounders were adjusted for and why they were included	11-12
		(*b*) Report category boundaries when continuous variables were categorized	11-12
		(*c*) If relevant, consider translating estimates of relative risk into absolute risk for a meaningful time period	n/a
Other analyses	17	Report other analyses done—eg analyses of subgroups and interactions, and sensitivity analyses	12-13
**Discussion**			
Key results	18	Summarise key results with reference to study objectives	14
**Limitations**			
Interpretation	20	Give a cautious overall interpretation of results considering objectives, limitations, multiplicity of analyses, results from similar studies, and other relevant evidence	14-16
Generalisability	21	Discuss the generalisability (external validity) of the study results	15-16
**Other information**			
Funding	22	Give the source of funding and the role of the funders for the present study and, if applicable, for the original study on which the present article is based	17

*Give information separately for cases and controls in case-control studies and, if applicable, for exposed and unexposed groups in cohort and cross-sectional studies.

Note: An Explanation and Elaboration article discusses each checklist item and gives methodological background and published examples of transparent reporting. The STROBE checklist is best used in conjunction with this article (freely available on the Web sites of PLoS Medicine at http://www.plosmedicine.org/, Annals of Internal Medicine at http://www.annals.org/, and Epidemiology at http://www.epidem.com/). Information on the STROBE Initiative is available at www.strobe-statement.org.

For peer review only
